# Immune and metabolic signatures characterise constipation-driven endophenotypes in Parkinson’s disease

**DOI:** 10.1038/s41531-025-01212-8

**Published:** 2025-12-20

**Authors:** Abbey Figliomeni, Samantha Winter, Madison Abonnel, Jade Kenna, Samantha Lodge, Luke Whiley, Andres Bernal, Jerome D. Coudert, Jonathan Noonan, Belinda Kaskow, Ryan Anderton

**Affiliations:** 1 Perron Institute for Neurological and Translational Science, Perth, WA Australia; 2 School of Biological Sciences, University of Western Australia, Crawley, WA Australia; 3 School of Health Sciences, University of Notre Dame Australia, Fremantle, WA Australia; 4 Australian National Phenome Centre, Health Futures Institute, Murdoch University, Perth, WA Australia; 5 Centre for Computational and Systems Medicine, Health Futures Institute, Murdoch University, Perth, WA Australia; 6 RESTORE Research centre, Université de Toulouse, CNRS, INSERM, EFS, Toulouse, France; 7 Personalised Medicine Centre, Health Futures Institute, Murdoch University, Perth, WA Australia; 8 Atherothrombosis and Vascular Biology Laboratory, Baker Heart and Diabetes Institute, Melbourne, VIC Australia; 9 School of Biomedical Sciences, University of Western Australia, Perth, WA Australia

**Keywords:** Diseases, Gastroenterology, Immunology, Neurology, Neuroscience

## Abstract

Parkinson’s disease (PD) is a progressive neurodegenerative disorder defined by motor impairments. However, people with PD (PwPD) experience a defined spectrum of non-motor symptoms, with gastrointestinal dysfunction the most common and earliest-presenting. Evidence suggests that PD pathology may originate in the gut, where microbial dysbiosis and immune dysregulation contribute to neuroinflammation, although mechanisms underlying this are unclear. PwPD (*n* = 31) and healthy controls (*n* = 28) were evaluated for clinical and gastrointestinal symptoms, faecal and plasma sample metabolomics, and comprehensive blood immunophenotyping. In PwPD, faecal samples exhibited reduced glutamate, succinate, and uracil concentrations, while plasma showed decreased 3-hydroxybutyrate and elevated creatine, succinate, and alanine levels. Immunophenotyping revealed a reduction in T cells, with evidence of altered effector capacity and functionality in CD4, CD8, MAIT and Vδ2 compartments. NK cells were expanded, while B cells were decreased in frequency with an enrichment of memory-like cells. Immune perturbations were correlated with levels of immunomodulatory metabolite succinate. Finally, clustering of blood parameters identified two PD endophenotypes distinguishable by gastrointestinal symptoms and T cell phenotypes associated with gut- and brain-tropism. These findings contribute to the growing understanding of metabolite-associated immune dysregulation in PD and highlight potential targets for early intervention in individuals presenting with gastrointestinal dysfunction.

## Introduction

Parkinson’s disease (PD) is a movement disorder characterised by the degeneration of dopaminergic neurons in the substantia nigra (SN). Currently, there are no effective preventative measures or treatment options for the disease, attributed to a lack of understanding of its pathophysiology. Despite being classified as a disease of the central nervous system (CNS), the knowledge that people with PD (PwPD) present with gastrointestinal (GI) dysfunction, particularly constipation, up to decades prior to the onset of motor symptoms has prompted research efforts into how this contributes to PD pathophysiology^1,2^. Such efforts led to the discovery of the presence of PD-defining neuronal inclusions of aggregated α-synuclein (Lewy pathology, LP) in the enteric nervous system of people with PD. Importantly, these features were found in samples obtained years prior to diagnosis and were associated with the severity of constipation in PwPD^3–6^. Based on these findings, Braak and colleagues proposed that PD may be initiated in the gut, now referred to as ‘gut-first’ PD, followed by progressive caudo-rostral appearance in the brainstem^7–9^. Many subsequent studies have supported elements of this hypothesis, yet the precise nature of the biological catalyst prompting the initiation of α-syn aggregation and promotion of its spread from the periphery to the brain remains unknown.

The discovery that PwPD present with an altered balance of gut microbes, known as dysbiosis, in conjunction with histological findings of a ‘leaky’ inflamed gut, and a significantly higher risk of PD development in those with GI inflammatory diseases, led to the proposal that chronic GI inflammation may play a key role in PD pathogenesis^10–16^. In this pathology model, the chronic production of bioactive molecules by a dysbiotic microbiome elicits gut mucosal inflammation, promoting eventual systemic immune dysfunction and facilitating the entry of inflammatory immune cells into the CNS, leading to the neuroinflammation-induced death of vulnerable dopaminergic neurons^17^. Indeed, immune dysfunction is now accepted as a core element of PD pathophysiology, with hypothesised involvement of immune cell subsets such as monocytes, monocytic myeloid-derived suppressor cells (M-MDSCs), natural killer (NK) cells, B cells, and T cells^18–20^.

Key pieces of this puzzle are missing, including how dysbiosis functionally impacts the immune system; which immune cells are initiators, bystanders, or protective against disease; and how these immune cells are triggered in a manner that differentiates the trajectory of PwPD from those with diseases such as GI disease or those with incidental Lewy body disease. This, in part, may be attributed to a lack of studies concomitantly assessing GI symptoms, metrics of dysbiosis, and blood immune cell dysfunction in PwPD. In addition, it is not understood why a small subset of PwPD do not adhere to this gut-first Lewy pathology spreading paradigm, and instead present with a rapid onset of brain-isolated pathology, referred to as ‘brain-first’ PD^8,21–23^.

To further understand the functional implications of dysbiosis and peripheral inflammation in PwPD, this study investigated small molecule metabolites in the faeces and plasma of PwPD and healthy controls (HCs) using nuclear magnetic resonance (1H-NMR) spectroscopy. In the same study cohort, comprehensive blood immunophenotyping was performed. This immunophenotyping further characterised immune populations identified as perturbed by previous PD studies, as well as the characterisation of unconventional T cell types including mucosal-associated invariant T (MAIT) cells, Vδ1 T cells, and Vδ2 T cells in PwPD, all of which play important roles in gut mucosal homeostasis. Faecal and plasma metabolite concentrations were correlated with PD-altered immune variables to explore potential interactions. Finally, blood-derived metrics were clustered to determine whether clinically relevant endophenotypes could be delineated within our PD group, revealing distinct immune-driven profiles that could only be explained by gastrointestinal symptom severity. Our findings build upon the existing knowledge of immune cell dysfunction in PwPD, prompt further investigation into immunopathology underlying ‘gut-first’ and ‘brain-first’ PD and highlight the importance of disease-stratification approaches to novel therapeutics.

## Results

### Participant characteristics

Participant characteristics are summarised in Table 1. There was no statistical difference between PD and HC study groups with regards to age, BMI, or male-to-female ratio. Consistent with previous findings^24^, PwPD exhibited significantly higher GSRS scores compared to HCs. Between-group comparisons of other lifestyle, health, and GSRS survey variables are shown in Table S2.

**Table 1:** Participant clinical characteristics

		PD (*n* = 31)	HC (*n* = 28)	*p-value*
***Age (years)***		65 + /− 9 [40 – 83]	63 + /− 11 [37 – 80]	0.37
***Sex (M/F)***		19/12	11/17	0.12
***Gastrointestinal Scale Rating Symptom (GSRS) total score***		6.5 + /− 3.8 [1 – 15]	4.2 + /− 3.7 [0 – 12]	0.019*
***Body Mass Index (BMI, kg/m***^2^***)***		26.1 + /−3.5 [20.0 – 33.6]	25.7 + /− 3.9 [19.9 – 34.8]	0.73
***Age at disease onset (years)***		58 + /− 12 [31 – 79]		
***Disease duration (years)***		8 + /− 8 [0 – 32]		
***Hoehn and Yahr Scale***	***Stage 1***	14		
	***Stage 2***	9		
	***Stage 3***	6		
	***Stage 4***	2		
***UPDRS Part III Score***		25.6 + /− 14.5 [7 – 62]		
***Levodopa Equivalent Daily Dose (LEDD, mg/day)***		800 + /− 655 [0 – 2632.5]		

Values presented as mean +/− standard deviation [range]. Parametric and non-parametric continuous variables were compared using Welch two-sample *t*-test or Wilcoxon rank sum tests, respectively. Categorical variables were compared using Fisher’s exact test. *p* < 0.05*.

### Increased microbial sensing capacity in classical monocytes and CXCR4-dependent tissue homing in M-MDSCs

**Fig. 1:**
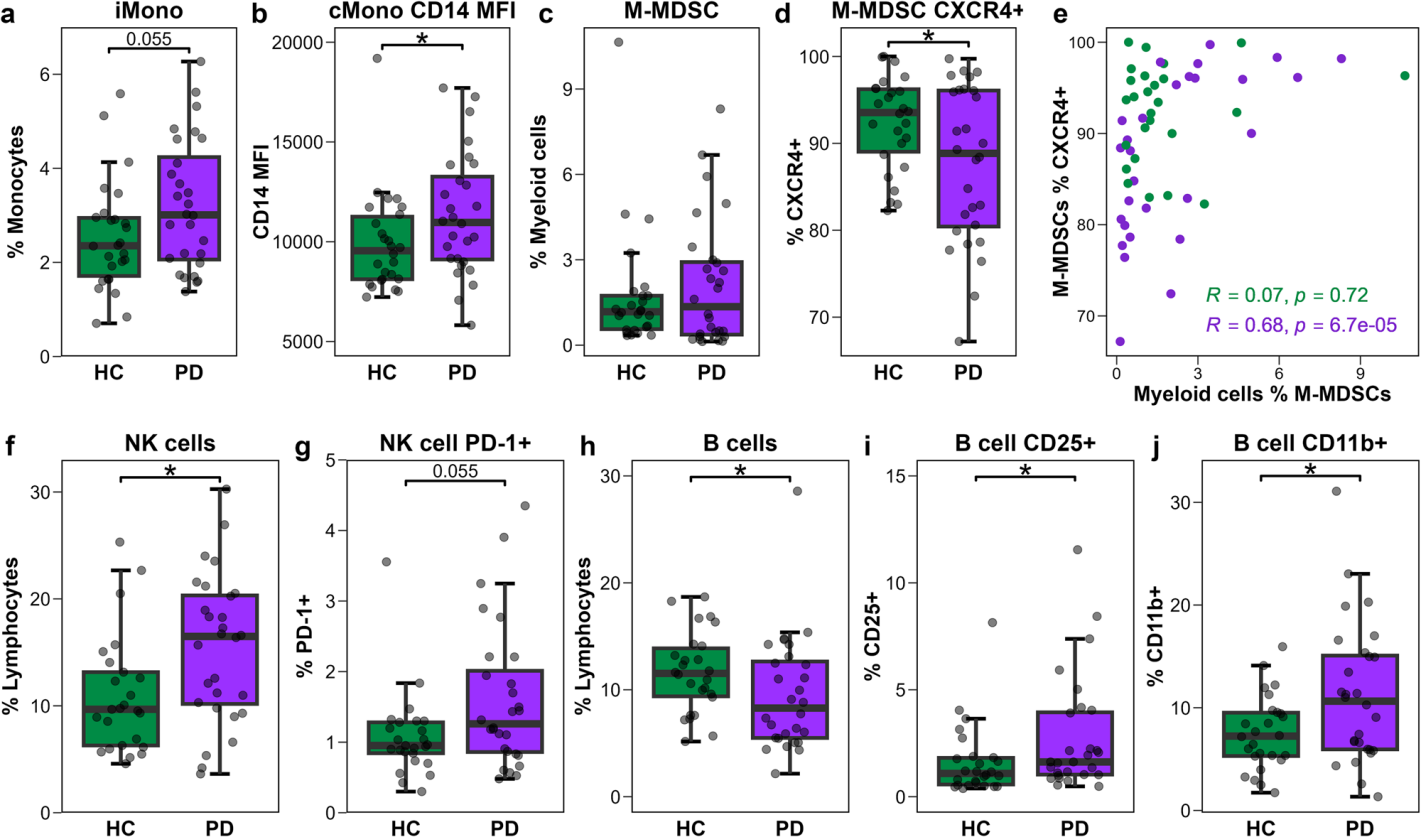
Phenotype signatures of monocytic, NK, and B cells. **a**−**d** Groupwise (*n* = 28 PD, *n* = 26 HC) boxplots showing: (**a**) the frequency of intermediate monocytes (iMono) among monocytes; (**b**) classical monocyte (cMono) CD14 expression by MFI; (**c**) the frequency of M-MDSCs among myeloid cells; and (**d**) the frequency of M-MDSCs expressing CXCR4. **e** Scatter plot showing groupwise Spearman correlations between M-MDSCs frequency and the percentage of M-MDSCs expressing CXCR4. **f**, **g** Groupwise (*n* = 28 PD, *n* = 25 HC) boxplots showing the frequency of: (**f**) NK cells among lymphocytes; (**g**) NK cells expressing PD-1. **h**, **i** Boxplots showing B cells among lymphocytes; (**i**) B cells expressing CD25; and (**j**) B cells expressing CD11b (*n* = 28 PD, *n* = 26 HC). Statistical comparisons performed by *t*-test or Wilcoxon rank-sum test, as appropriate. *p < 0.05 was considered statistically significant.

First, we comprehensively assessed immune phenotypes in the HC (*n* = 26) and PwPD (*n* = 28) participants. In the myeloid (CD33 + ) compartment, no difference in the overall frequency of monocytes, or frequency of classical and non-classical monocytes was observed in PwPD and HCs (Fig. S7a-c), however, there was a trend toward increased frequency of the intermediate monocyte subset (PD 3.0%, HC 2.4%; *p* = 0.055; Fig. 1a). A significant increase in the expression of microbial-sensing co-receptor CD14 (measured by MFI) on classical CD14^hi^ monocytes was seen in PwPD compared to HCs (Fig. 1b). M-MDSCs (defined as CD3-CD19-CD56-CD33 + HLA-DR^lo/neg^CD11b + CD14 + ) were unchanged in frequency among myeloid cells in PD subjects (Fig. 1c), however there was a significant decrease in the proportion of these cells expressing the peripheral tissue-homing marker CXCR4 (Fig. 1d). CXCR4 expression by M-MDSCs was correlated with their frequency in PwPD (*R* = 0.68, *q* = 0.002), but not in HCs (*R* = 0.07, *q* = 0.89; Fig. 1e), possibly suggesting their sequestration in peripheral tissues in PwPD.

### Increased NK cell frequency in PwPD

The overall frequency of NK cells, defined as CD3-CD19-CD14-CD56+ lymphocytes, was also assessed, revealing an increased frequency of NK cells in PwPD (Fig. 1f). The frequencies of the four predominant NK cell subsets (Fig. S1; CD56_bright_CD16-, CD56_bright_CD16 + , CD56_dim_CD16-, CD56_dim_CD16 + ) were unchanged in PwPD compared to HCs (Fig. S7d). To further characterise NK cell phenotypes, the expression of key regulatory receptors including NKG2D, PD-1 and TIM-3, were examined. No changes were observed in the expression of NKG2D or TIM3 (Fig. S7e, f), but a trending increase in the frequency of NK cells expressing inhibitory receptor PD-1 was observed in PwPD compared to HCs (Fig. 1g).

### Higher frequencies of CD25+ and CD11b + B cells in PwPD

The B cell compartment was also briefly assessed (Fig. S1). PwPD exhibited a reduced frequency of B cells among lymphocytes compared to HCs (Fig. 1h). Further phenotypic analysis revealed a significantly higher proportion of B cells expressing the activation marker CD25 (Fig. 1i). The frequency of B cells expressing the memory-associated marker, CD11b, was also increased in PwPD (Fig. 1j).

### Phenotyping and functional analysis of T cells reveals heightened activation and effector capacity in PD

**Fig. 2:**
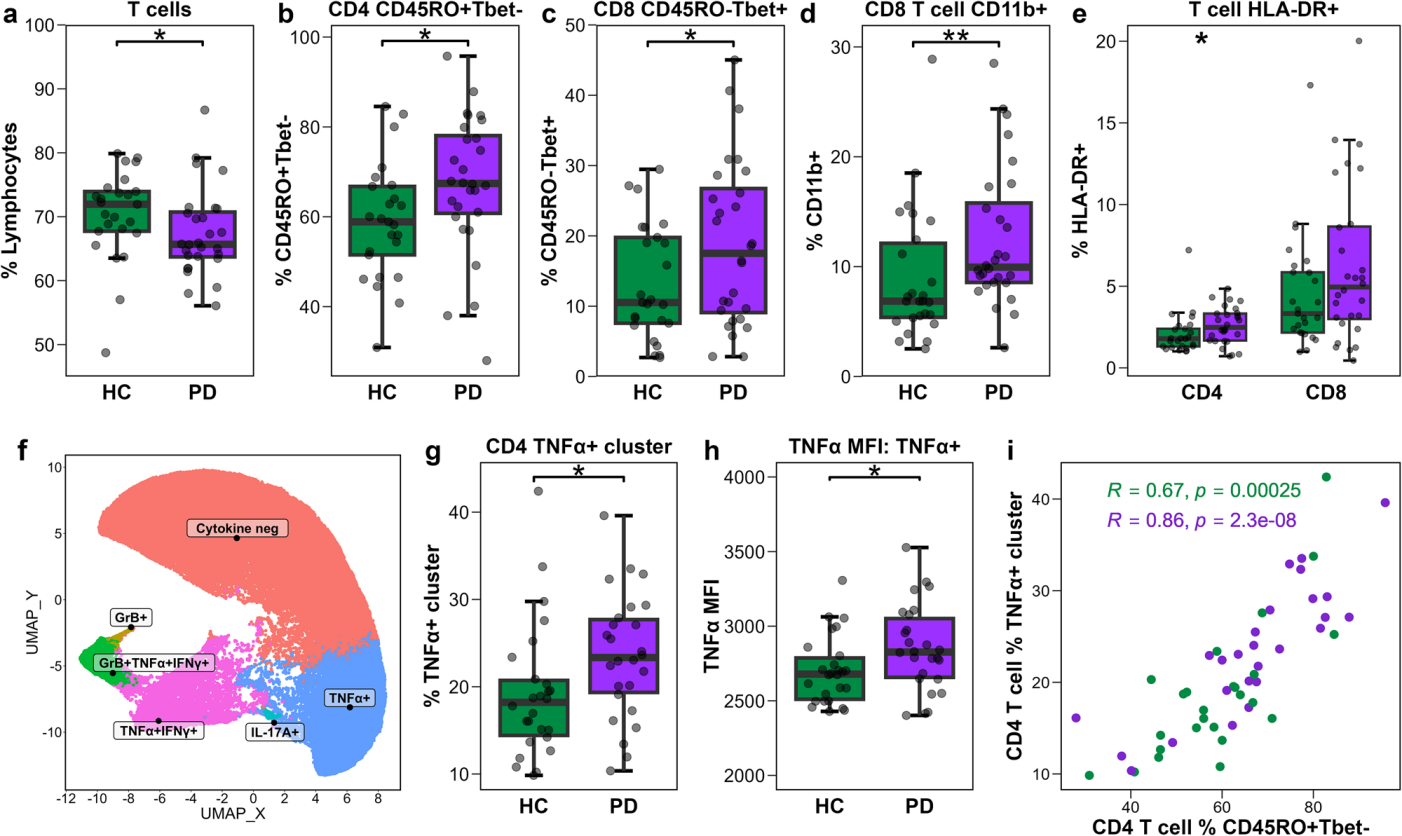
Analysis of T cell activation and effector functions. Boxplots showing frequency of: (**a**) T cells among lymphocytes; (**b**) CD45RO+Tbet- cells among CD4 T cells; (**c**) CD45RO-Tbet+ cells among CD8 T cells; (**d**) CD8 T cells expressing CD11b; and (**e**) HLA-DR+ cells among CD4 and CD8 T cells in PwPD (*n* = 28) and HCs (*n* = 25 (**a**−**c**, **e**; *n* = 26 d). **f** UMAP plot showing a random group-stratified subset of 100,000 stimulated CD4 T cells (50,000 PD and 50,000 HC), coloured by FlowSOM cluster identity. **g**, **h** Groupwise (*n* = 26 PD, *n* = 26 HC) boxplots showing: (**g**) TNFα + CD4 T cell cluster frequency among stimulated CD4 T cells; and (**h**) TNFα MFI of cells belonging to the CD4 T cell TNFα+ cluster. **i** Scatter plot showing Spearman correlation analysis between the frequencies of TNFα + CD4 T cells and of CD45RO+Tbet- CD4 T cells in each study group. Group comparisons were performed using the t-test or the Wilcoxon rank-sum test, as appropriate. *p < 0.05 was considered statistically significant.

T cells were assessed for abundance and phenotype changes using flow cytometry panels 1-3 (Figs. S1–3). PwPD exhibited a significant decrease in the frequency of T cells compared to HCs (Fig. 2a), although no differences were observed in the frequencies of CD4 or CD8 T cells (Fig. S8a).

Memory (CD45RO + ) CD4 T cells are frequently reported as enriched in PwPD^25–27^. In line with this, a trending increase in the frequency of CD45RO + CD4 T cells, was observed in PwPD compared to HCs, but no change was seen in CD45RO + CD8 T cells (Fig. S8b). Further analysis revealed this increase in CD45RO + CD4 T cells was predominantly driven by cells negative for transcription factor Tbet (Fig. 2b), suggesting the enrichment of a memory cell compartment in PwPD that does not conform to a Th1-associated effector paradigm.

An enrichment of terminal effector CD8 T cells has been described in PwPD^28,29^. In our study, we corroborate these findings and show a significant increase in the frequency of terminal effector-like CD45RO-Tbet+ CD8 T cells in PwPD (Fig. 2c). Likewise, expression of CD11b, a surrogate marker for recently activated effector CD8 T cells^30–32^, was also significantly increased in PwPD (Fig. 2d).

We next assessed the activation status of resting T cells from PwPD. We observed an increased frequency of HLA-DR + CD4 T cells in PwPD, but no change in CD8 T cells (Fig. 2e). The frequency of CD4 and CD8 cells expressing other activation- and exhaustion-associated markers CD25, CD69, PD-1, CD39, and TIM-3 was not significantly different between groups (Fig S8c,d).

Functional capacity of PMA/ionomycin-stimulated CD4 and CD8 T cells was interrogated using FlowSOM clustering (Fig S5). Clustering of stimulated CD4 T cells based on intracellular expression of TNFα, IFNγ, IL-17A, and granzyme B revealed six effector profiles: (1) cytokine-negative, (2) TNFα + , (3) IL-17A + , (4) TNFα + IFNγ + , (5) granzyme B + TNFα + IFNγ + , and (6) granzyme B+ (Fig. 2f). A significant increase in the frequency of the TNFα + CD4 T cell cluster was observed in PwPD (Fig. 2g). Furthermore, in PwPD CD4 T cells within this cluster exhibited a small increase in TNFα expression compared to HCs (Fig. 2h). Correlation of inter-panel variables revealed a strong association between the frequency of the TNFα + CD4 T cell cluster and that of CD45RO+Tbet- CD4 T cells, with significant positive correlations in both PwPD (*R* = 0.86, *q* < 0.0001) and HCs (*R* = 0.67, *q* = 0.006; Fig. 2i). No changes were observed in CD8 T cell cytokine polyfunctionality using the same clustering approach (Fig. S8e).

### MAIT cells exhibit an exhausted phenotype, reduced MR1 tetramer binding, and altered cytokine polyfunctionality PwPD

**Fig. 3:**
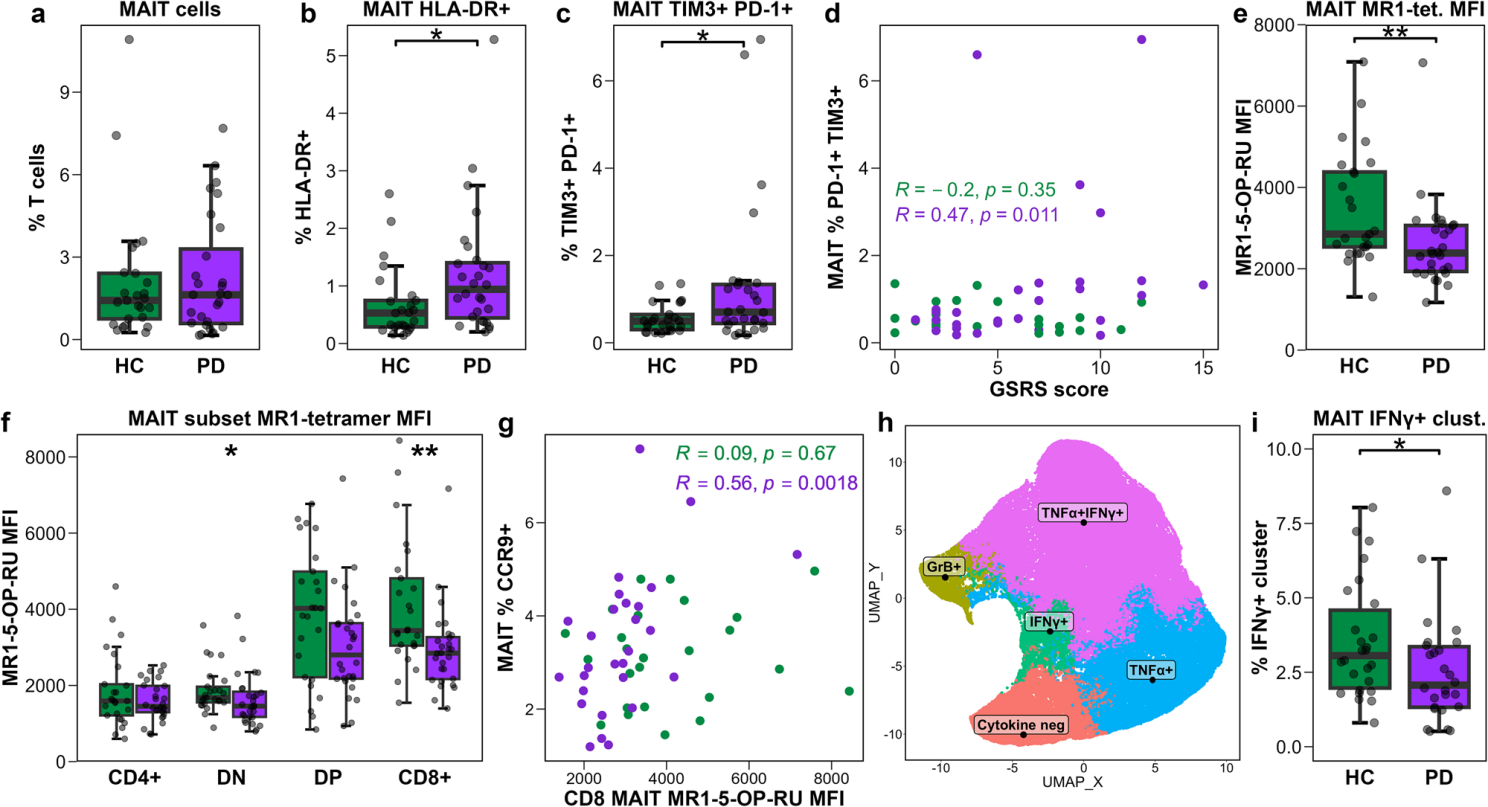
MAIT cells exhaustion- and activation-associated phenotypes, MR1 tetramer binding, and cytokine production. **a**−**c** Boxplots showing frequency of: (**a**) MAIT cells among T cells; (**b**) HLA-DR+ among MAIT cells; (**c**) TIM-3 + PD-1+ among MAIT cells. **d** Scatter plot showing correlation between the proportion of TIM-3 + PD-1 + MAIT cells and participant Gastrointestinal Symptom Rating Scale (GSRS) score. **e** Boxplots showing MAIT cell MR1-5-OP-RU tetramer MFI. **f** Boxplots showing MR1-5-OP-RU tetramer MFI on MAIT cell CD4/CD8 subsets. **g** Scatter plot showing Spearman-rank correlation between CD8 MAIT cell MR1-tetramer MFI and the frequency of CCR9 + MAIT cells. **h** UMAP displaying a random group-stratified subset of 100,000 stimulated MAIT cells, coloured according to FlowSOM cluster identity. **i** Boxplot showing the frequency of the IFNγ + MAIT cell cluster among stimulated MAIT cells. Boxplots and scatter plots are coloured according to study group, and comparisons between PD (*n* = 28 **a**−**g**; *n* = 26 **h**, **i**) and HC (*n* = 25 **a**−**g**; *n* = 26 **h**, **i**) groups performed by t-test or Wilcoxon rank-sum test, as appropriate. Statistical significance is indicated as *p < 0.05, **p < 0.01.

Studies in other dysbiosis-associated pathologies have revealed alterations in blood MAIT cell subset abundance, activation status, and cytokine polyfunctionality^33–35^. We performed the first phenotypical characterisation of MAIT cells, defined as MR1-tetramer positive T cells, in PwPD (Figs. S2,3). MAIT cell frequency among T cells was unaltered in PwPD compared to HCs (Fig. 3a). The relative abundance of MAIT cell subsets, defined by CD4 and CD8 expression, remained consistent across groups, with the expected predominance of CD4-CD8+ (CD8 MAIT) and CD4-CD8- double-negative (DN) MAIT cells (Fig. S9a)^36,37^. Expression levels of canonical MAIT cell transcription factors PLZF, EOMES, and RORγt were also similar between groups (Fig. S9b-d).

Next, the activation status of MAIT cells was assessed. A small but significant increase in HLA-DR + MAIT cells was observed in PwPD compared to HCs (Fig. 3b). Assessment of inhibitory receptors TIM-3 and PD-1 revealed a trend toward an increased frequency of TIM3 + MAIT cells (Fig. S9e), but no significant change in PD-1 expression, which was highly variable between participants (Fig S9f). Co-expression of inhibitory receptors by MAIT cells is observed in response to T cell receptor (TCR)-independent stimulation by mucosal inflammation-associated cytokines IL-12 and IL-18 and hence was assessed with respect to PD-1 and TIM-3. A significantly higher proportion of TIM3 + PD-1 + MAIT cells was detected in PwPD (Fig. 3c), a phenotype that is typically rare in the circulation in healthy individuals but is enriched in inflammatory conditions^33,38,39^. Notably, TIM3 + PD-1 + MAIT cells were present at frequencies above 1% in 11/28 of PD participants but only in 2/25 HCs. Moreover, the proportion of TIM3 + PD-1 + MAIT cells positively correlated with gut symptom severity, as measured by GSRS score, in PwPD (*R* = 0.47, *p* = 0.011) but not in HCs (*R* = -0.2, *p* = 0.35; Fig. 3d).

Since MAIT cell function is linked to MR1-5-OP-RU tetramer binding capacity^36^ MR1-tetramer binding was compared between groups. PwPD exhibited a significantly lower MR1-tetramer MFI compared to HCs (Fig. 3e). As MR1-tetramer staining intensity differs by MAIT cell subset due to the stabilising interaction of CD8 with MR1^36^, binding was analysed separately for each subset. A significant decrease in MR1 tetramer binding was observed in both CD8 MAIT cells and DN MAIT cells with a similar trend observed for CD4 + CD8+ (double-positive, DP) MAIT cells, but no change evident in CD4 MAIT cells (Fig. 3f). Notably, MR1-tetramer MFI positively correlated with the frequency of MAIT cells expressing intestinal homing chemokine receptor CCR9 in PwPD (*R* = 0.52, *q* = 0.089) but not in HCs (*R* = -0.01, *q* = 0.99) which was stronger when restricting the analysis to CD8 MAIT cell MR1-tetramer MFI (PD: *R* = 0.56, *q* = 0.039; HC: *R* = 0.09, *q* = 0.91; Fig. 3g).

Following this, MAIT cell functional capacity following PMA/ionomycin stimulation was assessed using FlowSOM clustering, as previously applied for CD4 and CD8 T cells. Based on TNFα, IFNγ, granzyme B, and IL-17A expression, stimulated MAIT cells were segregated into five effector profiles: (1) cytokine-negative, (2) TNFα+, (3) IFNγ+, (4) granzyme B + , and (5) IFNγ + TNFα + (Fig. 3h). A significant decrease in the frequency of the IFNγ + MAIT cluster was observed in PwPD (Fig. 3i), but no differences in the abundance of other clusters were detected (Fig S9g).

### Vδ2 T cells in PwPD exhibit a TEMRA phenotype skew and heightened pro-inflammatory cytokine production

**Fig. 4:**
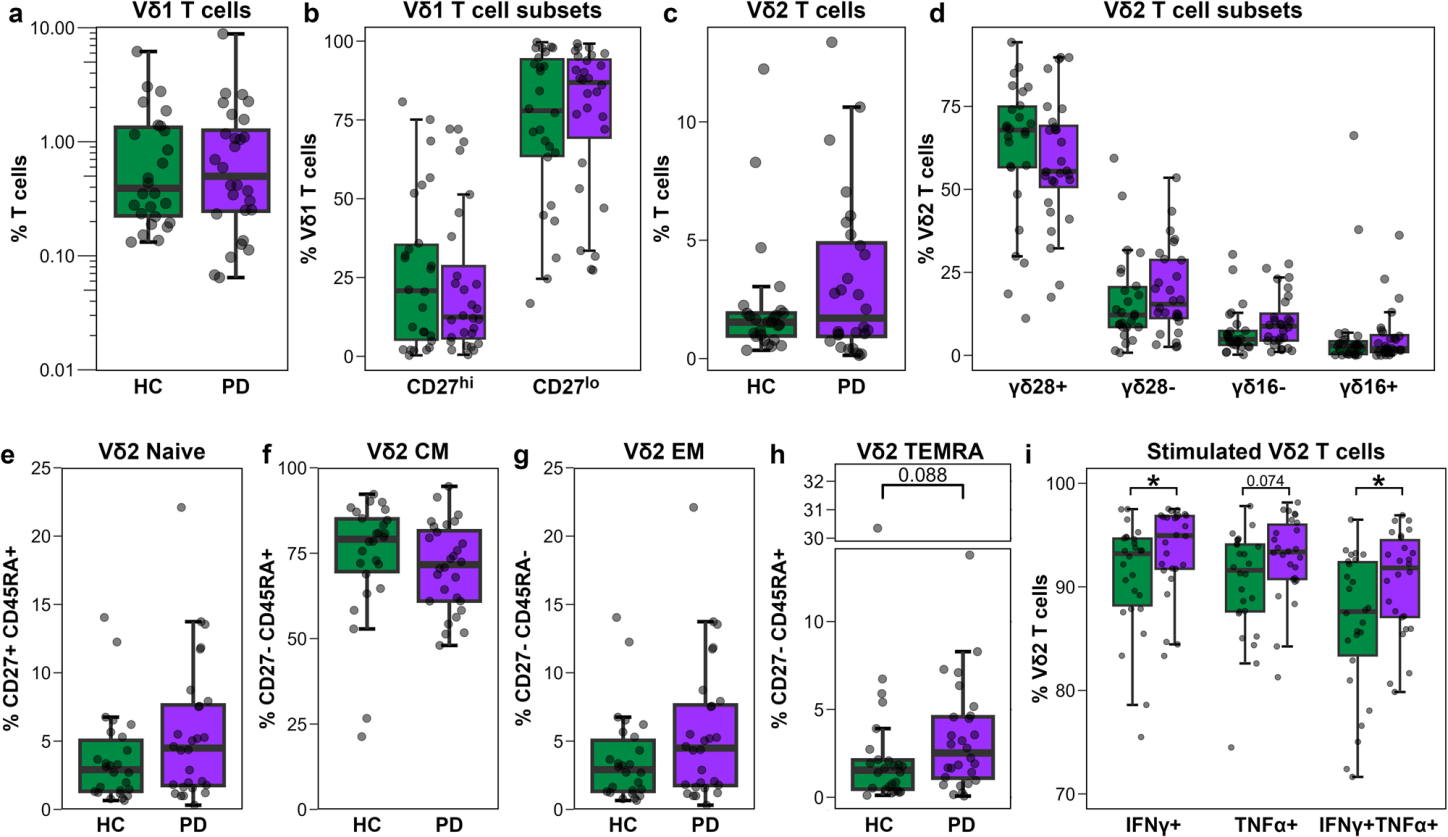
Phenotypic and functional characterisation of Vδ1 and Vδ2 T cells. **a**−**i** Groupwise (*n* = 28 PD, *n* = 26 HC) boxplots showing frequency of: (**a**) Vδ1 T cells among T cells; (**b**) CD27^hi^ and CD27^lo^ subpopulations among Vδ1 T cells; (**c**) Vδ2 T cells among T cells; (**d**) γδ28 + , γδ28-, γδ16, γδ16+ subpopulations among Vδ2 T cells; (**e**) CD27+ CD45RA+ ‘naïve’ among Vδ2 T cells; (**f**) CD27+ CD45RA- ‘central memory’ (CM) among Vδ2 T cells; (**g**) CD27- CD45RA- ‘effector memory’ (EM) among Vδ2 T cells; (**h**) CD27- CD45RA+ ‘TEMRA’ among Vδ2 T cells. **i** Groupwise (*n* = 26 PD, *n* = 26 HC) boxplots showing the frequency of stimulated Vδ2 T cells expressing IFNγ, TNFα, or both IFNγ and TNFα. Statistical comparisons performed by *t*-test or Wilcoxon rank-sum test, as appropriate. *p < 0.05 was considered statistically significant.

Previous PD studies have identified heterogeneous alterations in the frequency and phenotype of γδ T cells in PwPD. We hypothesised this heterogeneity may arise from the lack of delineation of the γδ T cell compartment into its two main subsets – Vδ1 and Vδ2 T cells – that are recognised to conform to distinct immunological paradigms. The γδ T cell compartment was assessed for overall abundance, then separately assessed for Vδ1 and Vδ2 T cells using validated phenotyping strategies^40,41^. The overall proportion of γδ T cells did not significantly differ between groups (Fig. S10). Similarly, the frequency of Vδ1 T cells was comparable between groups (Fig. 4a) and assessment of Vδ1 T cell phenotype according to CD27^hi^ (naïve) and CD27^lo^ (effector) subpopulations^41^ was also unchanged (Fig. 4b). Based on these findings, Vδ1 T cells appear unchanged in PwPD, and were not included in further functional analyses. Analysis of the innate-like Vδ2 γδ T cell subset, defined as Vγ9 + Vδ2 + T cells (Fig. S4), revealed the overall frequency was similar between groups (Fig. 4c). Division of the Vδ2 T cell compartment into four functionally distinct subpopulations based on CD28, CD27, and CD16 expression: CD28 + CD27 + CD16- (γδ28 + ), CD28-CD27 + CD16- (γδ28-), CD28-CD27-CD16- (γδ16-), and CD28-CD27-CD16+ (γδ16 + ) not reveal any differences between groups (Fig. 4d).

Vδ2 T cells can also be segregated into phosphoantigen (pAg)-induced differentiation state using naïve (CD27 + CD45RA + ), central memory (CM, CD27 + CD45RA-), effector memory (EM, CD27-CD45RA-), and CD45RA-expressing effector memory (TEMRA, CD27-CD45RA + ) designations^42,43^. No changes were observable in the frequency of naïve, effector memory, or central memory-like Vδ2 T cells, yet a trending increase was observed in the proportion of TEMRA-like Vδ2 T cells (Fig. 4e–h). We then assessed whether the TEMRA phenotype skew observed in Vδ2 T cells was associated with altered functional capacity. Since the vast majority of Vδ2 T cells co-expressed Vγ9 (median 98.8% PD, 99.0% HC), only the Vδ2 marker was used to define these cells in this analysis^44,45^. A small but significant increase in IFNγ-producing Vδ2 T cells was observed in PwPD, with a trend toward increased TNFα expression (Fig. 4f). Notably, the frequency of Vδ2 T cells co-expressing IFNγ and TNFα was significantly higher in PwPD than in HCs (Fig. 4f).

### Altered plasma small molecule metabolites concentrations correlate with disease duration and severity in PwPD

**Fig. 5:**
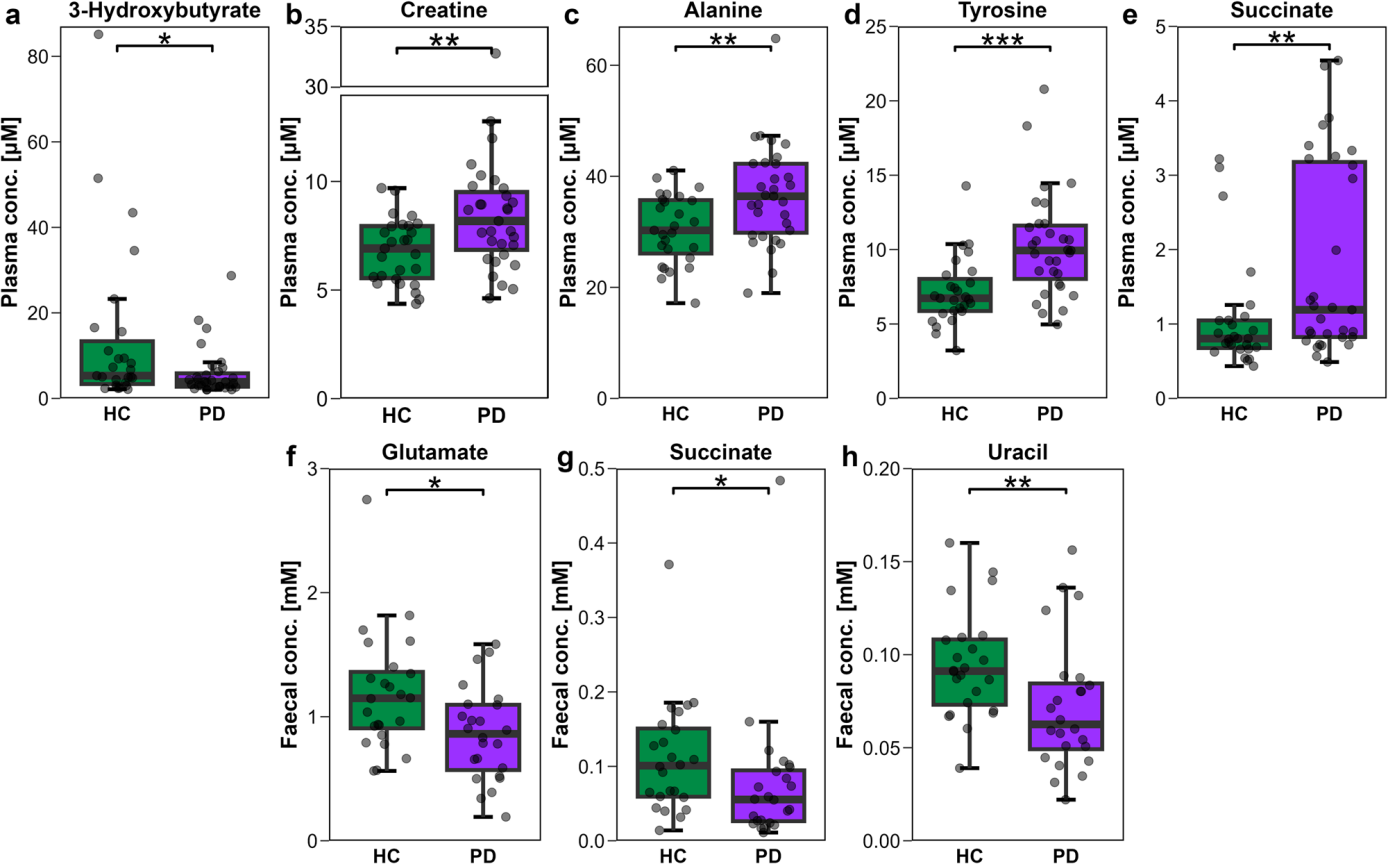
Altered plasma and faecal metabolite concentrations in PwPD versus HCs. **a**−**e** Groupwise (*n* = 31 PD, *n* = 27 HC) boxplots showing concentration (μM) of plasma metabolites (**a**) 3-hydroxybutyrate, (**b**) creatine, (**c**) alanine, (**d**) tyrosine, and (**e**) succinate. **f**−**h** Groupwise (*n* = 24 PD, *n* = 24 HC) boxplots showing faecal concentration (mM) of (**f**) glutamate, (**g**) succinate, and (**h**) uracil. Statistical comparisons were performed by Wilcoxon rank-sum or t-test, as appropriate, with significance indicated as *p < 0.05, **p < 0.01, ***p < 0.001.

Previous PD studies have identified inflammation-associated perturbations in plasma metabolites, although findings have been variable and not performed concomitantly with immunophenotyping. Using NMR, 17 metabolites were quantified in plasma samples collected from the same blood draw as the immune cells (Table S3). Relative to HCs, PwPD exhibited significant changes in the concentrations of five metabolites, with a decrease in 3-hydroxybutyrate (3HB); and increased concentrations of creatine, alanine, tyrosine, and succinate (Fig. 5a–e). To investigate potential clinical relevance, correlations between altered plasma metabolites and PD clinical metrics were assessed. In line with previous reports^46–48^, plasma tyrosine concentration was positively correlated with LEDD (R = 0.40, *p* = 0.027) while other metabolites showed no association.

### Decreased faecal concentration of glutamate, succinate, and uracil in PwPD

In addition to measuring plasma metabolites, we investigated faecal small molecule metabolite profiles in a subset of our study cohort of PwPD and HCs (Table S4). PwPD exhibited significantly lower faecal concentrations of glutamate, succinate, and uracil, compared to HCs (Fig. 5f–h).

### Plasma and faecal succinate concentration is correlated with pro-inflammatory immune metrics in a disease status-specific manner

**Fig. 6:**
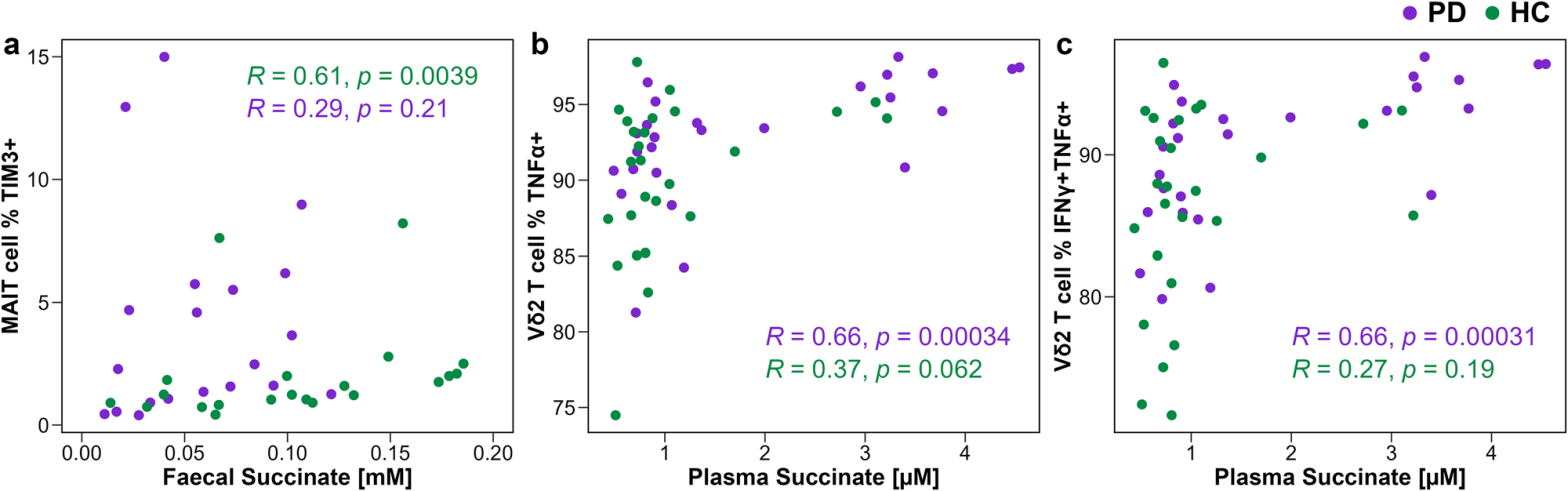
Prominent immune-metabolite correlations observed in study participants. **a**−**c** Scatter plots showing (where both metrics were performed) the correlation between: (a) faecal succinate concentration and frequency of TIM3 + MAIT cells (*n* = 21 PD*, *n* = 21 HC); (b) plasma succinate concentration and frequency TNFα + Vδ2 T cells (*n* = 26 PD, *n* = 25 HC); and (**c**) plasma succinate concentration and frequency IFNγ + TNFα + Vδ2 T cells (*n* = 26 PD, *n* = 25 HC). *0.48 mM PD group faecal succinate point removed for visualisation purposes, see Fig. 5g. Groupwise correlations performed by Spearman-rank, with correlation coefficients and p-values shown on plots.

To explore potential links between immune-modulating metabolites and immune cell phenotypes in PwPD, groupwise Spearman-rank correlation analyses were performed between PD group-altered plasma metabolites, faecal metabolites, and immune parameters. A total of 304 correlation pairs were computed for each study group (Fig. S11). The most noteworthy ( | R | > 0.5) correlations with faecal metabolites were observed exclusively in HCs, where faecal succinate concentration was positively correlated with the frequency of TIM3 + MAIT cells (*R* = 0.61, *p* = 0.0032, *q* > 0.1; Fig. 6a), and frequency of TIM3 + PD-1 + MAIT cells (*R* = 0.57, *p* = 0.0075, *q* > 0.1). However, the significance of these correlations did not survive FDR correction. For plasma metabolites, two correlations remained significant after FDR correction: higher plasma succinate concentrations correlated with a greater frequency of both TNFα-producing Vδ2 T cells (*R* = 0.66, *p* = 0.0003, *q* = 0.074; Fig. 6b), and IFNγ + TNFα + Vδ2 T cells (*R* = 0.66, *p* = 0.0002, *q* = 0.074; Fig. 6c) in PwPD.

### Clustering of PD group blood parameters reveals constipation-associated endophenotype in PwPD

To explore whether clinically relevant endophenotypes exist within our PD cohort, an unsupervised clustering approach was applied to blood-based immune and metabolic parameters. To conserve the maximum amount of biological information in the dataset, as well as to ensure all metrics are accessible at the level of blood sampling, only plasma metabolites (excluding levodopa-associated metabolite tyrosine) and immune cell phenotyping parameters were included. MFI metrics were removed to eliminate excessive collinearity. Where duplicate predictors exist (e.g. T cell frequencies), the most reliable metric, defined as the metric associated with the greatest number of exclusion markers, was retained. After filtering, the final dataset contained 166 predictors, including 150 immune population frequencies and 16 plasma metabolites.

K-means clustering of all predictors identified two clusters, which were overlayed on a PCA plot (Fig S12a). Despite the high parameter-to-observation ratio, the clusters exhibited clear separation along the first principal component (PC1). Inspection of the top contributors to PC1 revealed that the most influential variables were immune phenotyping metrics, while the highest-contributing metabolite, leucine, contributed minimally, ranking 51^st^ among all variables. To enhance cluster stability, the dataset was reduced to include the top 20 contributing variables (all immune) to PC1, after which clustering was re-performed (Fig S12b). This resulted in a 90.5% reduction of within-cluster variability (TWSS) and a 2.3-fold increase in cluster assignment confidence (SW).

**Fig. 7:**
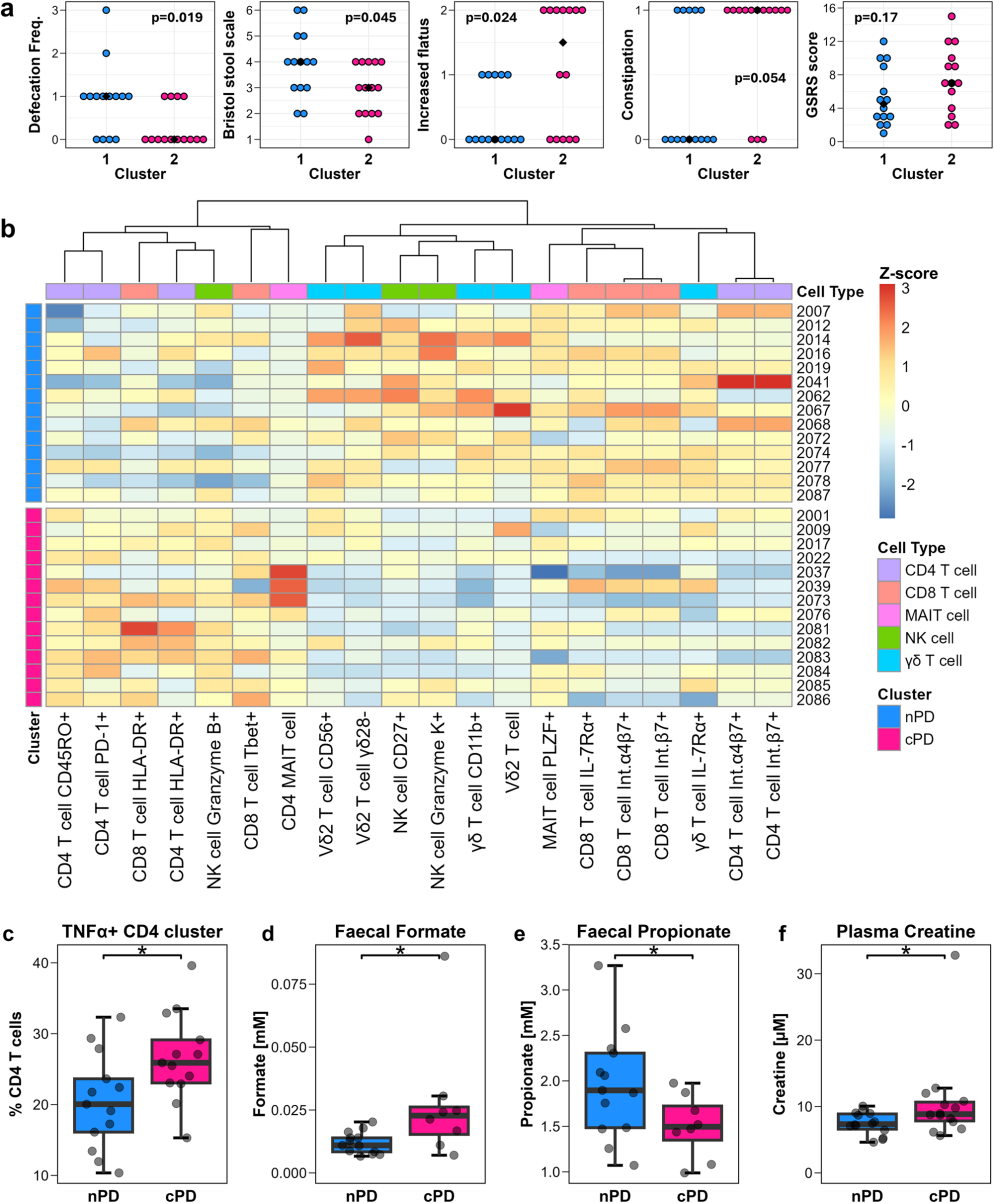
Investigation of clinical and biological parameters discriminant of PD endophenotypes. **a** Dot plots of clinical variables with the highest between-cluster discriminative capacity: GI survey ‘daily frequency of defecation’ [0 to 3, indicating less-than-once to 3 times per day], Bristol Stool Scale, GSRS ‘increased flatus’, self-reported presence of constipation yes[1]/no[0], GSRS scale total score. Medians for each cluster are indicated by black dots. **b** Heatmap of normalised immune variables used in clustering analysis, with rows labelled with PD participant identifier code and colour-coded according to cluster identity (nPD cluster blue, *n* = 14; cPD cluster pink, *n* = 14). Immune metrics columns are coloured according to immune cell type and ordered by hierarchical clustering. Heatmap colouring reflects relative upregulation (red + 3 SD) or downregulation (blue -3 SD) of immune metrics among PD participants. **c**−**f** Boxplots showing between-cluster differences in: (**c**) TNFα + CD4 T cell frequency, (**d**) faecal formate concentration, (**e**) faecal propionate concentration, and (**f**) plasma creatine concentration. Statistical comparisons of clinical variables were performed using Fisher’s exact test (binary response) or Wilcoxon rank-sum test (ordinal scale response). Between-cluster comparisons of biological parameters were performed by t-test or Wilcoxon rank-sum test according to variable distribution. *p < 0.05 was considered statistically significant.

To evaluate the clinical relevance of these immune-based clusters, demographic and GI survey variables were analysed. All statistically significant cluster-differentiating clinical variables were related to GI survey metrics (Fig. 7a). Participants assigned to Cluster 2 exhibited a lower self-reported defecation frequency, with most participants defecating less than once per day, (*p* = 0.019), lower Bristol stool scale score indicating harder stools (*p* = 0.045), and increased incidence of flatus (GSRS survey *p* = 0.024). Consistently, a trending difference was seen in the self-reported presence of constipation (*p* = 0.054), with most (11/14) Cluster 2 participants reporting “yes”, compared to 5/14 in Cluster 1. Although Cluster 2 participants exhibited a higher GSRS score, this did not reach statistical significance (*p* = 0.17), suggesting that between-cluster differences were primarily related to GI motility rather than other GI disturbances. Importantly, no significant differences were observed between clusters in terms of motor symptom severity (MDS-UPDRS III, *p* = 0.963), disease duration (*p* = 0.746), age (*p* = 0.280), sex (*p* = 0.704), or LEDD (*p* = 0.823). Together, we show that Cluster 2 represents a subgroup of PwPD with clinical features of decreased GI motility, including lower defecation frequency and harder stools. Based on this, the clusters were designated as nPD (low/no constipation) and cPD (PD with a higher incidence of constipation-associated metrics).

Despite some inter-individual heterogeneity, a distinct pattern in the up/down-regulation of hierarchically clustered immune variables defining the nPD and cPD clusters was evident (Fig. 7b). Notably, the variables driving cluster separation aligned with key immunological features, including T cells expressing gut-homing markers (integrin α4β7); T cell activation (HLA-DR) and exhaustion (PD-1); effector memory T cells (CD45RO, T-bet); NK cell maturity and cytotoxicity markers (CD27, granzymes B and K); reduced MAIT cell functionality (CD4 MAIT, lower PLZF expression); and an altered Vδ2 T cell compartment. Specifically, cPD participants exhibited a stronger T cell activation/exhaustion signature, an increased memory CD4 T cell population, a higher frequency of cytotoxic NK cells, and a greater abundance of CD4 MAIT cells. In contrast, nPD participants displayed greater frequencies of circulating integrin α4β7 + T cells, a striking increase in CD11b+ γδ T cells, a less mature/cytotoxic NK cell profile^49^, higher frequencies of IL-7Rα + T cells, and an expanded Vδ2 T cell compartment, likely driven by the γδ28- subset. Since neither stimulation panel variables nor metabolite concentrations were included in this clustering analysis, they were subsequently compared between cluster members where observations were available. Consistent with the increase in memory CD4 T cells, cPD participants exhibited a prominent increase in the frequency of TNFα + CD4 T cells (Fig. 7c). Additionally, the cPD cluster displayed higher faecal concentration of formate (Fig. 7d) and lower faecal concentration of short-chain fatty acid (SCFA) propionate (Fig. 7e) compared to the nPD cluster. Plasma creatine concentration was also elevated in cPD compared to nPD (Fig. 7f).

**Fig. 8:**
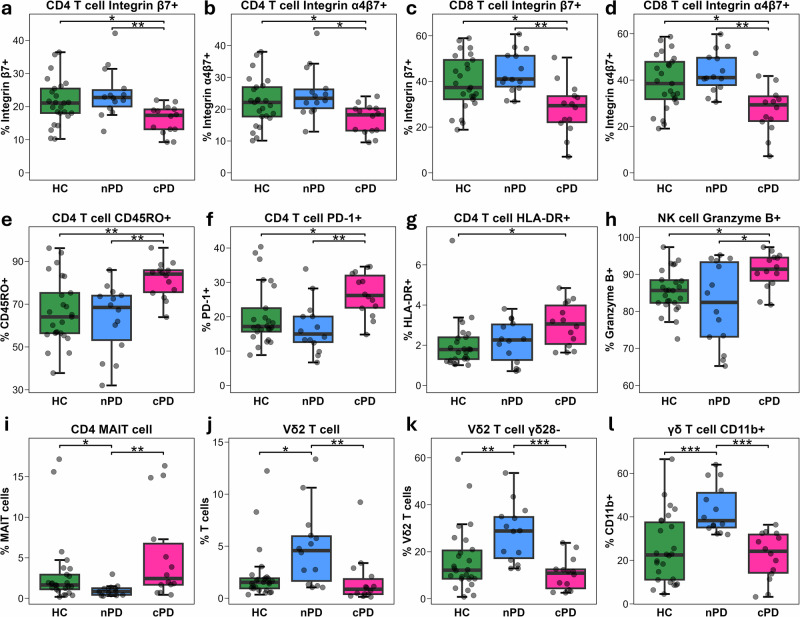
Frequency of immune variables used in clustering among PD endophenotypes and HC participants. Boxplots showing frequency (among parent cell population) of PD cluster-defining immune variables in PwPD grouped according to endophenotype (nPD: blue, *n* = 14; cPD pink, *n* = 14) and HCs (green, *n* = 25-26), including: (**a**) integrin β7 + CD4 T cells; (**b**) integrin α4β7 + CD4 T cells; (**c**) integrin β7 + CD8 T cells; (**d**) integrin α4β7 + CD8 T cells; (**e**) CD45RO + CD4 T cells; (**f**) PD-1 + CD4 T cells; (**g**) HLA-DR + CD4 T cells; (**h**) granzyme B + NK cells; (**i**) CD4 + CD8- MAIT cells; (**j**) Vδ2 T cells; (**k**) γδ28- Vδ2 T cell subset; and (**l**) CD11b + γδ T cells. Between-group comparisons performed using ANOVA or Kruskal-Wallis test, as appropriate. If significant, post-hoc Tukey’s HSD or Dunn’s test with Bonferroni correction for multiple comparisons was performed, respectively. Statistical significance is indicated as*p < 0.05, **p < 0.01, ***p < 0.001.

To further contextualise these cluster-based differences, we statistically compared the 20 cluster-defining immune variables between nPD, cPD, and HCs (Fig. 8). In both CD4 and CD8 T cell compartments, the frequency of integrin β7+ and integrin α4β7+ cells were significantly lower in the cPD group than in both the nPD cluster and HCs (Fig. 8a–d). Additionally, cPD participants exhibited significantly greater frequencies of CD45RO + CD4 T cells (Fig. 8e), PD-1 + CD4 T cells (Fig. 8f), and HLA-DR + CD4 T cells (Fig. 8g), consistent with a greater CD4 T cell activation signature compared to the nPD cluster and HCs. In addition, a significantly greater frequency of NK cells expressed granzyme B in the cPD cluster compared to nPD and HCs (Fig. 8h).

In contrast, CD4 MAIT cells were lower in frequency in nPD than in both HCs and cPD (Fig. 8i). Additionally, nPD participants exhibited a significantly increased frequency of Vδ2 T cells, whereas cPD participants displayed Vδ2 T cells frequencies similar to HCs (Fig. 8j). The same pattern was observed in the frequency of γδ28- Vδ2 T cells (Fig. 8k), indicating this phenotype may be driving the expansion observed. Most prominently, CD11b expression on γδ T cells was increased in nPD compared to both cPD and HCs (Fig. 8l). Differences in other cluster-defining variables with respect to HCs were less prominent (Fig. S13).

## Discussion

Peripheral blood immune dysfunction is a feature of PD pathophysiology and may be intricately linked to PD risk-associated GI comorbidities. This study provides further support for this association, demonstrating altered phenotypes across multiple immune cell types, including monocyte-derived cells, NK cells, B cells, CD4/CD8 T cells, MAIT cells and Vδ2 T cells in PwPD. CD4, MAIT, and Vδ2 T cells from PwPD also exhibited altered functional capacity, providing further support for the role of chronic inflammation in PD pathogenesis. Furthermore, to the best of our knowledge, this is the first comprehensive immunophenotyping study to concomitantly assess blood and faecal metabolomic composition within the same PD cohort. Quantification of metabolic phenotypes revealed PD-specific alterations in host and microbial metabolism, many of which were in alignment with the existing literature. Importantly, we identified an association between host- and microbial-derived succinate and distinct immunophenotypes in PwPD, highlighting a potential metabolic-immune axis relevant to PD pathology. Finally, unsupervised clustering of blood-based immune parameters revealed two PD endophenotypes that were clinically distinguished only by gastrointestinal motility symptoms and were best delineated by T cell subsets relevant to gut- and brain-homing behaviour.

Our study provides further evidence to support the notion of altered monocyte subset distribution and phenotype in PwPD. We found no differences in monocyte frequency between PwPD and HCs but did observe increased CD14 expression on classical monocytes in PwPD. As a key microbial-sensing receptor, CD14 plays a crucial role in detecting pathogen-associate molecular patterns such as endotoxin and oxidised phospholipids^50,51^. Considering PwPD exhibit increased gut permeability and oxidative stress^11^, the elevated CD14 expression could reflect chronic activation of classical monocytes, the monocyte subset most primed for innate sensing^52,53^. M-MDSC are a subset of myeloid cells derived from chronically activated monocytes, with phenoconversion usually taking place in the pathogenically inflamed tissue, such as the gut^54^. While we saw no difference in M-MDSCs between PwPD and HCs, others have reported either an increase or a tendency toward an increase^18,55^. The decreased CXCR4 expression on M-MDSCs, which was positively correlated with the frequency of M-MDSCs, is particularly interesting given the implication of the CXCL12-CXCR4 axis in PD pathogenesis. CXCL12 is elevated in the blood of PwPD^56^, and two independent studies have reported increased CXCR4 and CXCL12 expression in post-mortem SN tissue from PwPD^57,58^ These findings suggest that CXCR4-mediated immune cell trafficking may contribute to PD progression, and the role of M-MDSCs warrants further investigation.

Our finding of increased NK cells abundance in PwPD is consistent with previous studies^59–63^, including a recent report showing NK cells were increased both in frequency and absolute count in early (<2 years post-diagnosis) PD^59^. In our study, the frequency of NK cells expressing the immune checkpoint PD-1 was trending towards an increase in PwPD. PD-1 upregulation on NK cells has been associated with reduced cytotoxic capacity in chronic inflammatory conditions such as infection and cancer^64–68^, suggesting a possible impairment of NK cell function in PD.

The reduced frequency of B cells in PwPD found in our study is consistent with multiple reports indicating lower B cells abundance in PD^27,69–74^. Scott, et al.^72^ found that lower B cell frequencies were associated with more severe motor impairment and increased risk of cognitive decline, suggesting a potential protective role for B cells in PD. Our finding of an increased frequency of CD25 + B cells in PwPD could have multiple interpretations. While CD25+ expression has been used to define regulatory B cells (Bregs)^75,76^, previous studies have reported conflicting results regarding Breg dynamics in PD, with some studies reporting increased^73^, and others decreased^72,77^ frequencies in PwPD relative to HCs. Alternatively, CD25 may suggest elevated B cell activation in PwPD^78^, consistent with the well-documented notion of immune activation in PD^79^. CD11b is a β2 integrin predominantly expressed by memory B cells^80^ and its elevated frequency in PwPD supports prior findings of memory B cell accumulation in PD^81,82^. Notably, CD11b has also been shown to mark a specific subset of gut mucosa-tropic B cells, accumulating in the colon of mice with colitis and humans with inflammatory bowel disease (IBD), where they serve as primary IgA producers and were shown to elicit a protective role^83^. This raises the intriguing possibility that the decreased circulating B cell frequency in PwPD may result from their migration to inflamed gut tissues, a hypothesis that warrants further investigation.

Collectively, our study both corroborates and extends finding of T cell phenotype and functional dysregulation in PD, supporting the notion that the T cell compartment is depleted, with an enrichment of memory cells and heightened activation and effector capacity^79^. The decreased frequency of T cells among circulating lymphocytes is consistently reported^27,28,60,61,63,84–86^, and it is speculated that this observation may be attributed to altered homing behaviour with T cell infiltration into the PD brain^87–89^. Findings from our study also align with previous studies, with increased frequencies of HLA-DR+ and CD45RO+ (memory) CD4 T cells^25–27,90^. A noteworthy finding was the strong correlation between frequencies of CD45RO+Tbet- memory CD4 T cells and the TNFα + CD4 T cell cluster, suggesting that downregulation of the Th1-driving transcription factor Tbet in these cells may allow TNFα production over IFNγ production. TNFα is elevated in the gut mucosa, blood, and CNS of PwPD^91–93^; and is associated with PD genetic-risk^94–96^. Additionally, people with IBD taking anti-TNF therapy have been shown to exhibit a 78% reduced risk for developing PD^97^. Our finding of an increased frequency of CD4 T cells with enhanced TNFα production capacity suggests these cells may contribute to the elevated TNFα levels reported in PwPD^98^. However, it remains unclear whether the accumulation of Tbet-CD45RO + CD4 T cells is a consequence of PD-specific antigenic stimulation, or a broader ‘inflammageing’-associated phenomenon in PD^20,70^.

Another intriguing observation was elevated expression of CD11b on CD8 T cells in PwPD. CD11b + CD8 T cells are thought to represent a pool of antigen-specific T cells with potent effector activity^30–32^ and may have relevance to CD8 T cell infiltration in the brain of PwPD^88^. Additionally, our study validates findings from, Capelle, et al.^28^ found an increased frequency of effector CD45RO-Tbet+ CD8 T cells in the blood of PwPD. While our phenotyping data cannot definitively determine the terminal differentiation status of these cells, prior studies found that CD45RO- CD8 T cells in PwPD are predominately CD45RA + , alongside a concomitant expansion in TEMRA CD8 T cells^28,29^.

A higher proportion of MAIT cells with an activated and exhausted phenotype, altered cytokine production, and reduced MR1-tetramer binding capacity were seen in PwPD. Our findings align with those observed in other chronic inflammatory pathologies, including IBD, type 1 diabetes, and colorectal cancer^33–35^. In IBD, MAIT cells are depleted in the circulation, upregulate activation and exhaustion markers, and show accumulation in inflamed colonic tissue^34,99^. A similar mechanism may be at play in PD, where an enrichment of TIM3 + PD-1 + MAIT cells could be the consequence of GI inflammation. This hypothesis is supported by our finding that our finding that TIM3 + PD-1 + MAIT cell frequency correlated with gut symptom severity. MAIT cells with high MR1-tetramer affinity are typically more responsive to antigenic stimulation, including a greater capacity to produce MAIT-associated cytokine IFNγ^36^. The strong negative correlation between MR1-tetramer MFI and CCR9 + MAIT cell frequency in our study suggests that high-affinity MAIT cells may preferentially home to the gut in PwPD. Interestingly, in other pathologies associated with GI inflammation, IFNγ-skewed MAIT cells are similarly decreased in the circulation^35^ and enriched in the colon^33^. In addition to its immunomodulatory effects, IFNγ is implicated in important mucosal homeostasis functions such as the promotion of mucous secretion by goblet cells to protect the intestinal epithelium^100^. Future studies should investigate whether gut-resident MAIT cells in PwPD exhibit an altered TCR repertoire and effector capacity compared to circulating subsets, and whether altered barrier defence function may exacerbate or protect against gut-brain pathology in PD.

Our findings extend prior studies on γδ T cell dysregulation in PD, refining this paradigm to be exclusive to the Vδ2 T cell subset. TEMRA Vδ2 T cells represent pAg-experienced cells with a low proliferative capacity, poor lymph node representation, and high cytotoxic potential^45,101,102^. The tendency towards an enriched TEMRA Vδ2 compartment PwPD suggests chronic antigenic stimulation, potentially driven by gut-derived microbial pAgs. Supporting this, gut microbiota composition has been shown to modulate the Vδ2 T cell compartment^103^. Vδ2 T cells from PwPD also displayed elevated pro-inflammatory cytokine production capacity following in vitro stimulation. Whether this more pro-inflammatory state is driven by microbial-derived pAg stimulation, and whether these cells contribute to neurodegeneration by pro-inflammatory cytokine production in the CNS of PwPD should be addressed by future studies.

Plasma metabolic phenotyping revealed alterations in creatine, alanine, 3HB, and succinate aligning with a metabolic signature indicative of mitochondrial dysfunction and oxidative stress^104^. Creatine, a critical component of the phosphocreatine system for ATP buffering^105^, was also elevated in PwPD. A single PD study quantified creatine and reported no changes^106^, however, elevated creatine has also been demonstrated in vivo in the brain of PwPD using proton magnetic resonance spectroscopy^107^. Alanine, a non-essential α-amino acid involved in protein synthesis and cellular energy generation^108^ was increased in PwPD. One study similarly reported elevated plasma alanine levels in PD^109^, although other studies have reported no change^47,106^ or did not quantify alanine^110–113^. Notably, Figura, et al.^114^ found plasma alanine concentrations were highest in PwPD with shorter disease duration and lower symptom severity (<3 years disease duration, H&Y stages 1-2), consistent with our higher representation of H&Y stages 1-2. 3HB is the primary ketone body synthesised in the liver and constitutes the brain’s primary source of energy when glucose is absent^115^. 3HB is also implicated in immune modulation, histone deacetylation inhibition, and oxidative stress protection^116^. Despite this, findings pertaining to 3HB in PwPD are sparse. One study found that plasma 3HB levels were decreased in individuals who were later diagnosed with PD^117^, whereas, other studies have reported an increase in plasma 3HB^118^ or no change^106^ relative to HCs. Given the potential neuroprotective role for 3HB indicated by PD preclinical models^119,120^, it is important for future PD metabolomic studies to elucidate the relationship between 3HB concentration and disease stage in PwPD. Furthermore, the cellular specificity and pathophysiological relevance of mitochondrial disfunction in PwPD should be interrogated in future studies using approaches that combine in vitro functionality and mitochondrial function, such as a Seahorse assay following autologous co-culture of sorted immune cells and induced pluripotent stem cell-derived dopaminergic neurons^121,122^.

Succinate, a key TCA cycle intermediate and immunomodulatory metabolite, was elevated in plasma but reduced in faecal samples from PwPD. Pathan, et al.^106^ similarly found plasma succinate to be increased in PD, and that elevated succinate was one of two common metabolic alterations among people with atypical parkinsonian disorders (multiple system atrophy and progressive supranuclear palsy) compared to HCs. However, other studies have reported no change^123^ or a decrease in plasma succinate in PwPD^111^. Interestingly, we observed a bimodal plasma succinate concentration distribution in PwPD, with one subset exhibiting markedly elevated succinate levels, and another with levels comparable to HCs. Correlation analyses revealed this subset of ‘succinate-high’ PD participants exhibited increased Vδ2 T cell pro-inflammatory cytokine production capacity. Given that succinate (via the inhibition of succinate dehydrogenase) accumulates under conditions of chronic inflammatory stress^124–128^ and dysregulation of microbial innate immune defence pathways such as the IRG1-itaconate axis^129,130^, elevated succinate may reflect a heightened inflammatory state in PwPD. Succinate is also recognised as an immunomodulatory metabolite, acting in a paracrine-like manner through succinate receptor 1 and reinforcing its own accumulation in a feed-forward loop^131,132^. Whether elevated plasma succinate accumulates consequential to or is a driver of pro-inflammatory cytokine production by Vδ2 T cells should be explored by future functional studies. Conversely, our study found decreased faecal succinate concentration in PwPD, consistent with a prior PD faecal metabolomics study^133^. The apparent discordance between plasma and faecal succinate levels likely reflects their distinct sources with plasma succinate largely host-derived, whereas faecal succinate is predominantly derived from microbial metabolism^134,135^. The decrease in faecal succinate concentration may reflect alterations to the relative abundance of succinate-producing (e.g. the *Prevotellaceae* family) and succinate-consuming bacteria in the dysbiotic gut microbiome of PwPD^131,136^.

Investigation of other faecal metabolites revealed decreased concentrations of glutamate and uracil in PwPD, possibly be reflective of the functional consequences of microbial dysbiosis and/or increased nutrient demand by enterocytes. Glutamate, a key excitatory neurotransmitter in the enteric and central nervous systems^137,138^, was significantly reduced in PwPD. Previous studies have reported similar findings^133,139^ raising the question as to whether this finding is a result of disease-specific processes such as increased uptake by enterocytes or perturbations to glutamate-producing or glutamate-consuming metabolic pathways in colon symbionts. A meta-analysis of PD gut microbiome studies^14^ predicted an enrichment of the glutamate degradation pathway in PD-associated microbiota, potentially driven by an increased abundance of glutamate-consuming bacterial genera, such *Akkermansia*, in the microbiome of PwPD. Uracil, one of the four main nucleobases used in RNA synthesis, was also significantly reduced in faecal sample from PwPD. Uracil can be synthesised endogenously by human cells such as enterocytes^140^, but dietary sources and gut microbiota also contribute to its availability^141^. The decrease in faecal uracil may reflect either increased host utilisation due to intestinal inflammation or dysbiosis-driven alterations in microbial uracil metabolism. Future gut microbiome sequencing studies with concomitant quantification of uracil in the faeces of PwPD are required to understand the significance of this finding.

Finally, unsupervised clustering of all resting immune frequency and plasma metabolite parameters identified two immune parameter-defined PD endophenotypes, which were distinguished only by GI survey metrics. While this is a pilot investigation, requiring validation in larger independent validation cohorts, the finding that the 20 most significant cluster-discriminating immune parameters were centred GI homeostasis and CNS trafficking is striking. This pattern closely aligns with the ‘gut-first’ and ‘brain-first’ PD subtypes proposed by Horsager, et al.^8^. Although the clinical evidence from our study is much more subjective in nature, the identification of self-reported measures of slower colonic transit time in the cPD cluster, despite no significant differences in motor symptom severity or other potential confounders (age, sex, LEDD), is intriguing. Given that our cohort comprised relatively mild-severity PD participants, this may represent the first evidence of immune-faecal biomarkers discriminating ‘gut-first’ and ‘brain-first’ PD subtypes.

The most distinctive cluster-discriminating immune variables were CD11b+ γδ T cells and integrin α4β7 + T cells, which were drastically increased in nPD (no/low constipation) and decreased in cPD (PD with higher incidence of constipation-associated metrics), respectively. Unfortunately, CD11b was only quantified in γδ T cells overall. However, since Vδ2 T cells predominate the peripheral γδ T cell compartment and given that similar trends were observed for γδ T cell and Vδ2 T cell cluster-defining parameters, nPD is likely characterised by alterations in the γδ T cell isolated to Vδ2 T cells. Given the role of CD11b in CNS infiltration, the nPD cluster may be associated with Vδ2 T cells primed for CNS migration. In contrast, cPD participants exhibited a depletion of integrin α4β7 + T cells relative to both nPD and HCs. In conjunction with the higher prevalence of GI dysfunction, this phenotype could be suggestive of gut-homing sequestration in response to dysbiosis-driven GI inflammation^142^ Additionally, cPD participants displayed a reduced frequency of circulating CD4 MAIT cells, which are known to exhibit decreased TCR-dependent cytokine production capacity and PLZF expression comparted to other MAIT subsets^36,37^. Future studies should determine whether these immune shifts reflect a systemic manifestation of pathology-driven tissue trafficking or a broader immune dysfunction driving PD pathology.

Further supporting a gut-inflammatory signature in cPD, TNFα-skewed CD4 T cells were significantly more abundant in cPD. This raises the possibility that PwPD who have constipation or broader GI dysfunction may benefit more from TNF-lowering therapeutic interventions. Additionally, faecal propionate levels were decreased while formate levels were increased in cPD. Propionate, a SCFA produced by homeostatic gut microbes fermenting insoluble carbohydrates, promotes immune tolerance, and is known to be decreased in constipation and IBD^131,143–145^. Conversely, higher faecal formate could be a metabolic consequence of a shift toward protein fermentation over carbohydrate fermentation, a common occurrence in chronic constipation and dysbiotic gut environments^131,146,147^. While our subgroup analysis was limited by sample size, these trends suggest that faecal metabolites may serve as useful indicators of PD-related gut dysbiosis.

Given that T cell infiltration has been shown to precede LP^88^, it is tempting to speculate that the ‘brain-first’ and ‘body-first’ PD subtypes may actually represent distinct immunopathologies. If LP accumulation occurs in a region-specific manner as a consequence of these immune differences, this could explain the variable clinical trajectories observed in PwPD. Future immunological studies, particularly in early PD and at risk populations (e.g. iRBD) should seek to differentiate ‘body-first’ and ‘brain-first’ subtypes by employing a similar clustering method on larger sample sizes^8^. Additionally, these findings also underscore the need for differential therapeutic approaches based on PD subtype. For example, interventions currently being trialled in PwPD, such as faecal microbial transplant^148,149^ or vagus nerve stimulation^150–152^, may have drastically different efficacy depending on aetiology and LP distribution. Importantly, there are currently no accessible prodromal markers to identify ‘brain-first’ PD. The presence of elevated CD11b+ γδ T cells in nPD raises the intriguing possibility that this subset could serve as an early biomarker of ‘brain-first’ subtype.

This study has some limitations. Of note, the sample size was modest, and all inferences made on PD progression were cross-sectional, limiting the ability to assess longitudinal immune or metabolic changes. As this small-cohort study was considered a pilot investigation, univariate comparison adjustment for false discovery rate was not performed, and findings require validation in larger independent cohorts. In particular, the markers distinguishing the cPD cluster, should be validated in people with ‘body-first’ PD-associated conditions, such as idiopathic rapid eye movement sleep behaviour disorder and incidental Lewy body disease to determine whether these immune and metabolic alterations can better identify at-risk individuals. Regarding the metabolomic analysis, the use of a 1H-NMR-based platform restricted the detection of only a subset of small molecule metabolites. As a result, key metabolic pathways relevant to PD pathophysiology may have been underrepresented. Future paired immune-metabolomic studies utilising other platforms, such as mass spectrometry, could provide a more comprehensive metabolic profile. Finally, faecal metabolomic analyses were not complemented by gut microbial composition profiling, and future studies will be needed to determine whether the observed metabolic changes in PwPD correspond to alterations in specific microbial taxa.

Currently available therapeutics for PwPD are only palliative, and do not halt disease progression. Given that GI dysfunction and its associated comorbidities can emerge years before PD diagnosis, a critical question in PD research is why some individuals with this phenotype progress to develop PD, while others do not. Additionally, the pathophysiological mechanisms underlying distinct ‘brain-first’ and ‘body-first’ patters of LP distribution in PD remain poorly understood. Our findings, along with prior research, suggest that the immune system may play a key role in shaping these disease trajectories. Future longitudinal studies applying multi-omics approaches to paired gut and blood samples from high-risk individuals and PwPD are essential to identify potentially modifiable immune-metabolic pathways and develop targeted interventions for PD prevention and treatment.

## Methods

### Participant recruitment and assessment

Study participants (*n* = 31 PD, *n* = 28 HC) were recruited between 2018 and 2021 from the Perron Institute Movement Disorders Clinic (Nedlands, WA) and Parkinson’s disease Western Australia support groups, as previously described^153^. All PD participants had a confirmed diagnosis of idiopathic PD by a movement disorders neurologist, based on the United Kingdom Brain Bank criteria. HCs were primarily the spouses, relatives, or caregivers of PD study participants. Human Research Ethics approval for participant recruitment and biological sample collection was granted by the University of Western Australia (approval number RA/4/20/4470) and all participants provided written informed consent. Participants were excluded based on predefined criteria designed to minimise confounding factors known to influence immune, GI, or neurological health. Exclusion criteria included: history of GI disease (inflammatory bowel disease, small intestinal bacterial overgrowth, *H. pylori* infection); concurrent chronic autoimmune, or systemic inflammatory disorders; morbid obesity (BMI > 35 kg/m^2^); type 1 or type 2 diabetes; antibiotic use within the past three months; major surgery within the past three months; chronic immunosuppressive therapy; or a history of neurological disease (for HCs).

During recruitment, participants completed a confidential questionnaire assessing disease history, medication use, environmental risk factors, and GI health. GI health was assessed using the validated Gastrointestinal Symptom Rating Scale (GSRS) questionnaire designed to evaluate the frequency and severity of GI symptoms^154–156^. Each of the 15 questions are graded on a 4-point Likert scale (0 – no occurrence of symptom, to 3 – frequent symptom occurrence), and tallied to give a total severity score indicative of the overall level of GI dysfunction. GSRS subcategories (abdominal pain, dyspeptic symptoms, indigestion, bowel dysfunction) were analysed to assess GI dysfunction by anatomical region (upper/low GI tract) or pathology-specific features such as constipation^24^.

As anti-parkinsonian medications vary in composition, half-life, and blood brain barrier permeability, their total levodopa equivalent daily doses (LEDD) values were computed using established conversion formulas^157^. Motor symptom severity in PwPD was assessed using the Hoehn and Yahr (H&Y) staging^158^ and part III of the Movement Disorders Society Unified Parkinson’s Disease Rating Scale (MDS-UPDRS III)^159^. To ensure consistency between ratings, all assessments of PD participants were assessed during their medication ‘ON’ state by the same trained practitioner.

### Biological sample collection and quality control

Venous whole blood (40 mL) was collected from each participant into heparin-coated collection tubes and processed within four hours of collection. Peripheral blood mononuclear cell (PBMC) isolation was performed using SepMate™ tubes following the manufacturer’s protocol. Whole blood was mixed with an equal volume of phosphate-buffered saline (PBS) + 2% foetal bovine serum (FBS) and layered onto Lymphoprep™ separation medium and centrifuged (21 °C, 20 min, 1200 g, gradual deceleration). Plasma was aliquoted into five 2 mL cryovials per participant and snap-frozen at -80 °C. The PBMC layer was transferred, washed, and cryopreserved in 90% FBS, 10% dimethyl sulfoxide in isopropanol-based freezing containers, then transferred to liquid nitrogen for long-term storage. One HC participant was unable to provide a blood sample on the day of assessment (risk of vasovagal syncope) and only provided a faecal sample. One HC participant and two PD participants could only provide sufficient blood for three and four out of five flow cytometry experiments, respectively. Four PBMC samples were excluded from flow cytometric analysis due to either poor PBMC separation quality or cell viability (<90%). Final participant numbers for all flow cytometry experiments are shown in Table 1.

A subset of study participants elected to provide faecal samples for metabolomic analysis by NMR (*n* = 24 PD, *n* = 24 HC, Table S1). Participants were provided with a sterile faecal sample collection kit with printed instructions detailing home collection of the sample: participants were to collect faecal sample into a sterile collection pot and store temporarily at -18 °C until collection by a research team member for transport to the laboratory -80 °C freezer where it was stored until processing. Faecal samples were processed into faecal water samples, as per the standard operating procedure adapted from^160,161^. Briefly, faecal samples were thawed at 4 °C, resuspended in a 1:2 ratio (g/mL) with ultra-pure water, then homogenised. Samples were then centrifuged, and the clear supernatant (‘faecal water’) extracted and stored at -80 °C until analysis.

### Flow cytometry

PBMCs were immunophenotyped using five multicolour flow cytometry panels, with gating and antibody details shown in supplementary figures 1-5. Panels 1-4 (phenotyping) and Panel 5 (stimulation) were performed in batches of 12 and 18 participants, respectively, balanced according to disease status. Batch effects were controlled for using BD FACSDiva^TM^ software application settings, linked to Cytometer Setup and Tracking (CS&T) beads (BD Biosciences) that were run within an hour of acquisition. Batch alignment was verified using clustering analyses of pooled pre-processed live events, visualised on dimensionality-reduced space (Fig. S6).

PBMCs were thawed in groups and rested in RPMI supplemented with 10% FBS and 1% penicillin/streptomycin in a 37 °C incubator for one hour (phenotyping panels) or 12 hours (stimulation panel). Stimulated samples received eBioscience™ Cell Stimulation Cocktail and Protein Transport Inhibitor Cocktail before a further 4 hour incubation. After incubation, cells were washed, stained with viability dye (Zombie^TM^ UV or FVS-575), then surface marker antibodies and either fixed in 2% PFA for 10 minutes (Panel 3) or in eBioscience^TM^ fixation-permeabilization reagent. If applicable, cells were also stained with an intracellular marker antibody cocktail. Stained samples were immediately acquired using a BD LSRFortessa^TM^ cytometer, at the UWA Centre for Microscopy Characterisation and Analysis, Nedlands, Western Australia.

### Proton nuclear magnetic resonance (^1^H NMR) spectroscopy

For NMR analysis, plasma was thawed at 4 °C and prepared using 300 µL plasma mixed with 300 µL phosphate buffer (75 mM Na_2_HPO_4_, 2 mM NaN_3_, 4.6 mM TSP in D_2_O, pH 7.4) and added to 5 mm outer diameter SampleJet NMR tubes following the recommended protocol for in vitro analytical and diagnostic procedures^162^. Faecal water samples were thawed at 4 °C before preparation by centrifugation and resuspension in potassium phosphate buffer (1.5 M KH_2_PO_4_, 2 mM NaN_3_, 0.1% TSP, pH 7.4). Samples were then vortexed before transfer to 5 mm SampleJet tubes (Bruker Biospin GmbH, Ettlingen, Germany).

All NMR analyses were performed on a Bruker 600 MHz Advance III HD spectrometer equipped with a 5 mm BBI probe and fitted with the Bruker SampleJet robot cooling system set to 5 °C. Automation of the acquisition procedure was controlled by Bruker IconNMR Software. A full quantitative spectrometer calibration was completed prior to analysis^160^. Sample analyses were completed at 310 K for the plasma samples and 300 K for the faecal samples.

Plasma samples underwent a series of NMR experiments performed according to Bruker’s In Vitro Diagnostics research method set. This comprised three experiments performed in automation: first, a ^1^H 1D experiment with solvent presaturation (32 scans, 98 304 data points, spectral width of 18028.85 Hz), a 1D Carr-Purcell-Meiboom-Gill (CPMG) spin-echo experiment (32 scans, 73 728 data points, spectral width of 12019.23 Hz), and a 2D J-resolved experiment (2 scans with 40 t1 increments).

For the faecal samples, two experiments were completed in automation, comprising the standard 1H 1D experiment with solvent presaturation (pp: noesygppr1d, 32 scans, 65 K data points, spectral width of 12019.23 Hz) and a 2D J-Resolved experiment (pp: jresgpprqf, 2 scans with 40 t1 increments).

### ^1^H NMR spectra processing and metabolite quantification

Metabolites were manually assigned using the Australian National Phenome Centre databases and published literature^163^. The identification of 17 and 19 metabolites was confirmed and quantified in plasma and faecal samples, respectively. For metabolite quantification, targeted resonances were modelled as combinations of pseudo-Voigt profiles and fitted to the experimental spectrum using a Levenberg-Marquardt optimisation algorithm and a polynomial baseline. Overlapped signals were optimised together. Goodness of fit was initially assessed through simple heuristics based on residuals and correlations. Integrals of the validated models were computed using the ‘full width at half maximum’ approximation and transformed to molar concentrations using the ERETIC internal standard^164^ and calibration procedure, part of the Bruker’s In Vitro Diagnostics research method set. In plasma samples, the quantification process was performed both on the standard 1D experiment and on the CPMG, and the consistency of the results was corroborated by correlation analysis. This redundancy provided analytical cross-validation and discarded potential errors from overlapping resonances of plasma proteins. The concentrations used for statistical analyses were those derived from the CPMG experiment. Model optimisation was implemented in R using the *minpack.lm* package, and model visualisation was implemented in JavaScript using EPFL’s visualiser (https://github.com/npellet/visualizer, https://my.cheminfo.org/).

To correct for faecal metabolite concentration variation associated with faecal water content, faecal metabolite concentration data were normalized using probabilistic quotient normalisation, implemented using the *Metaboanalyst* software companion R package^165^ with the reference spectrum chosen as the average of all HC samples, as has been implemented in previous faecal NMR metabolomic studies^166,167^. The dilution factor of plasma samples due to the presence of the Lymphoprep^TM^ reagent was consistent across samples, and as such no post-quantification normalisation was necessary.

### Statistical analysis

All statistical analyses were conducted using R (version 4.3-4.4). Summary statistics for parametric and non-parametric data (percentage change and fold-change, FC) were calculated with reference to the group mean or median, respectively. Flow cytometry data processing and analysis were performed using FlowJo software, in conjunction with the R packages *CytoML* and *FlowWorkspace*. Median Fluorescence Intensity (MFI) metrics were computed using the FlowWorkspace package, while clustering of flow cytometry events was performed using the *Spectre* R package.

Between-group differences were assessed using Fisher’s test for categorical variables. For numerical variables, groupwise normality was first assessed by the Shapiro-Wilk test, followed by either a two-tailed Wilcoxon rank-sum or *t*-test, as appropriate, with *p* < 0.05 considered significant. Correlations between pairs of immune variables, metabolites, and clinical variables were performed using the Spearman rank-sum correlation test. Multiple correlation analyses were adjusted for false discovery rate (FDR) using the Benjamini-Hochberg method^168^, with *q* < 0.1 considered significant. Boxplots and scatterplots were generated using the *ggplot2*^169^, *ggpubr*^170^, *ggtext*^171^, and *ggbreak*^172^ R packages.

For multivariate analysis, combined blood parameter data (immune cell frequencies and plasma metabolites) were standardised (mean-centred and scaled to unit variance) to ensure equal weighting of predictors. Principal component analysis (PCA) was performed using the singular value decomposition method in R, visualised using the *factoextra*^173^ R package. Clustering of PwPD blood immune and metabolite variables was performed using the K-means method. Cluster stability and the optimal number of clusters were determined using the total within cluster sum of squares (TWSS) and silhouette width (SW). For multiple group comparison of immune variables, pooled residual distributions were assessed before applying Kruskal-Wallis or ANOVA as appropriate. If statistically significant (*p* < 0.05), post-hoc comparisons were performed using Dunn’s test or Tukey’s HSD test, respectively, with Bonferroni correction applied for multiple comparisons. Heatmaps were generated using the *Pheatmap*^174^ package, with hierarchical clustering of rows or columns indicated by dendrograms and performed using the complete linkage method.

## Supplementary information

Supplementary information

## Data Availability

The datasets generated during this current study will be made available upon reasonable request.

## Code availability

The underlying code for this study is not publicly available but may be made available to qualified researchers upon reasonable request to the corresponding author.
