# Supplementary material for: Immune and metabolic signatures characterise constipation-driven endophenotypes in Parkinson’s disease
Source: NPJ Parkinsons Dis. 2025 Dec 20;12:2. doi: 10.1038/s41531-025-01212-8 (PMC12764879; doi:10.1038/s41531-025-01212-8)
Supplement: Supplementary file 1 — Supplementary information [file 41531_2025_1212_MOESM1_ESM.pdf]

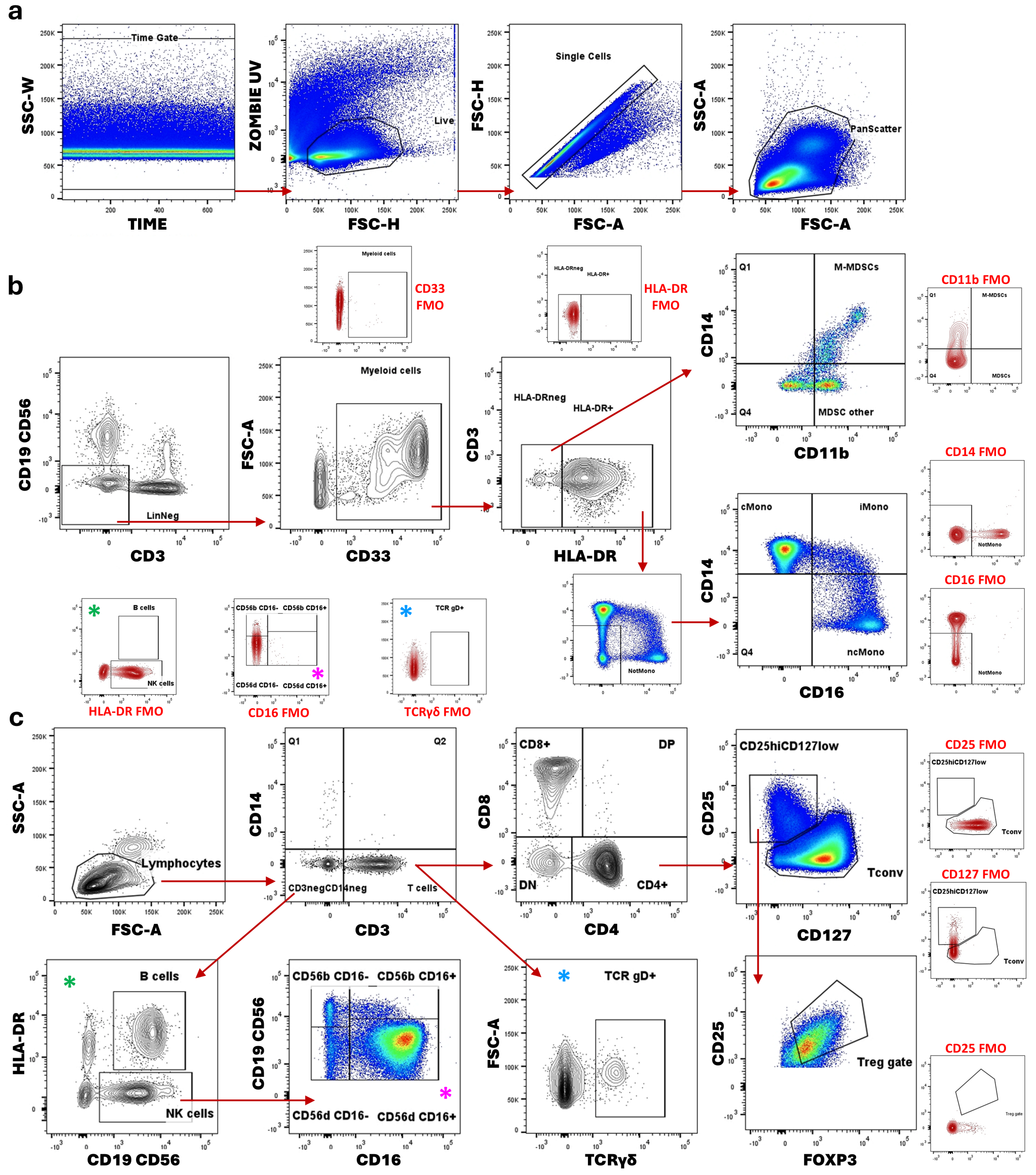

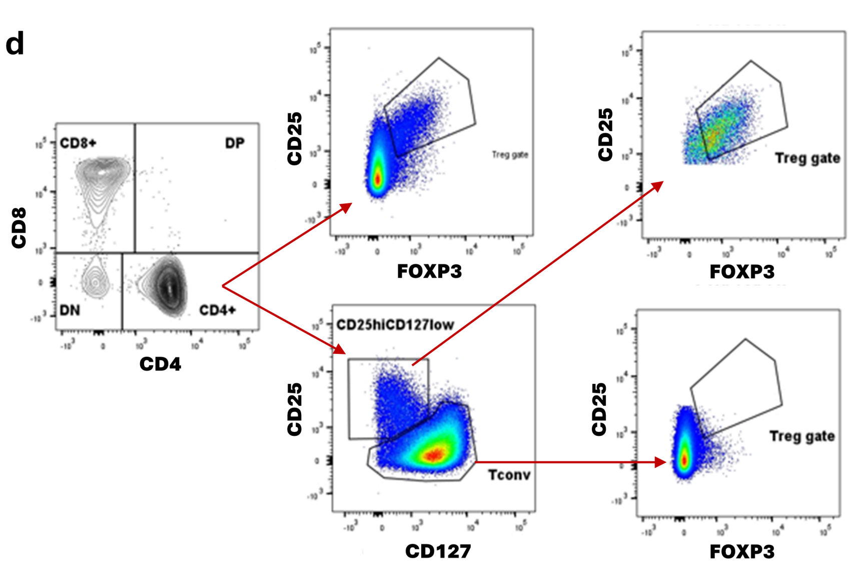

e

Antigen	*Fluorochrome*
CD3	BUV737
TCRγδ	PE-Cy7
CD8α	APC-Cy7
CD4	AF700
FOXP3	AF647
CD25	BV785
CD127	PE/Dazzle 594
CD39	BV605
CXCR4	BV421
CD14	PE-Cy5
CD16	BV711
HLA-DR	KIRAVIA Blue
CD11b	BV510
CD33	PE
CD56<br>CD19	BUV395
Viability	Zombie™ UV

Figure S1. Flow cytometry Panel 1 antibody details and gating strategy. *Representative gating for flow cytometry Panel 1, 'Broad immunophenotyping'. (a) Preprocessing of flow cytometry events by first eliminating any technical artefacts in the time dimension (if applicable), then gating live cells, singlets, and lymphocytes and monocytes based on size. (b) Myeloid cell (monocytes, M-MDSCs) gating strategy, from events in 'pan-scatter' gate. (c) T cell (including CD4 T cells, CD8 T cells, Tregs, and γδ T cells), NK cell, and B cell gating strategy, from events within the 'lymphocyte' gate. (d) Treg gating strategy justification, demonstrating a lack of conventional T cells (T_{conv}) within the Treg gate. (e) Panel antibody details. Fluorescence minus one (FMO) controls shown as red contour plots adjacent to corresponding gates or otherwise indicated for relevant plot with a coloured asterisk (*). Abbreviations: M-MDSC, monocytic myeloid-derived suppressor cell; ncMono, non-classical monocyte; iMono, intermediate monocyte; cMono classical monocyte; Treg, regulatory T cell.*

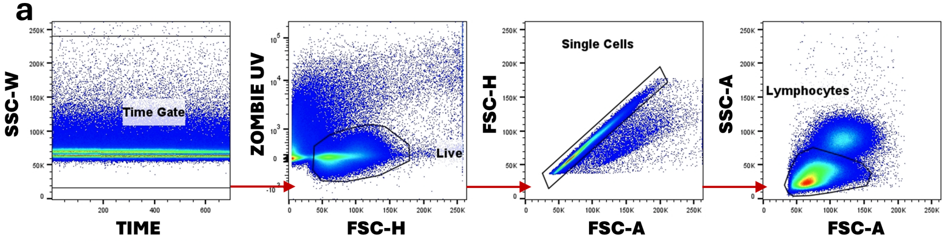

b

Antigen	Fluorochrome
MR1-5-OP-RU	PE
CD3	BUV737
CD4	AF700
CD8	APC-H7
CD161	BV786
CD45RO	BUV395
CD49d	BV421
Integrin β7	FITC
Eomes	PE-Cy7
PLZF	AF647
RORγt	PE-eFluor610
Tbet	BV605
Viability	Zombie™ UV

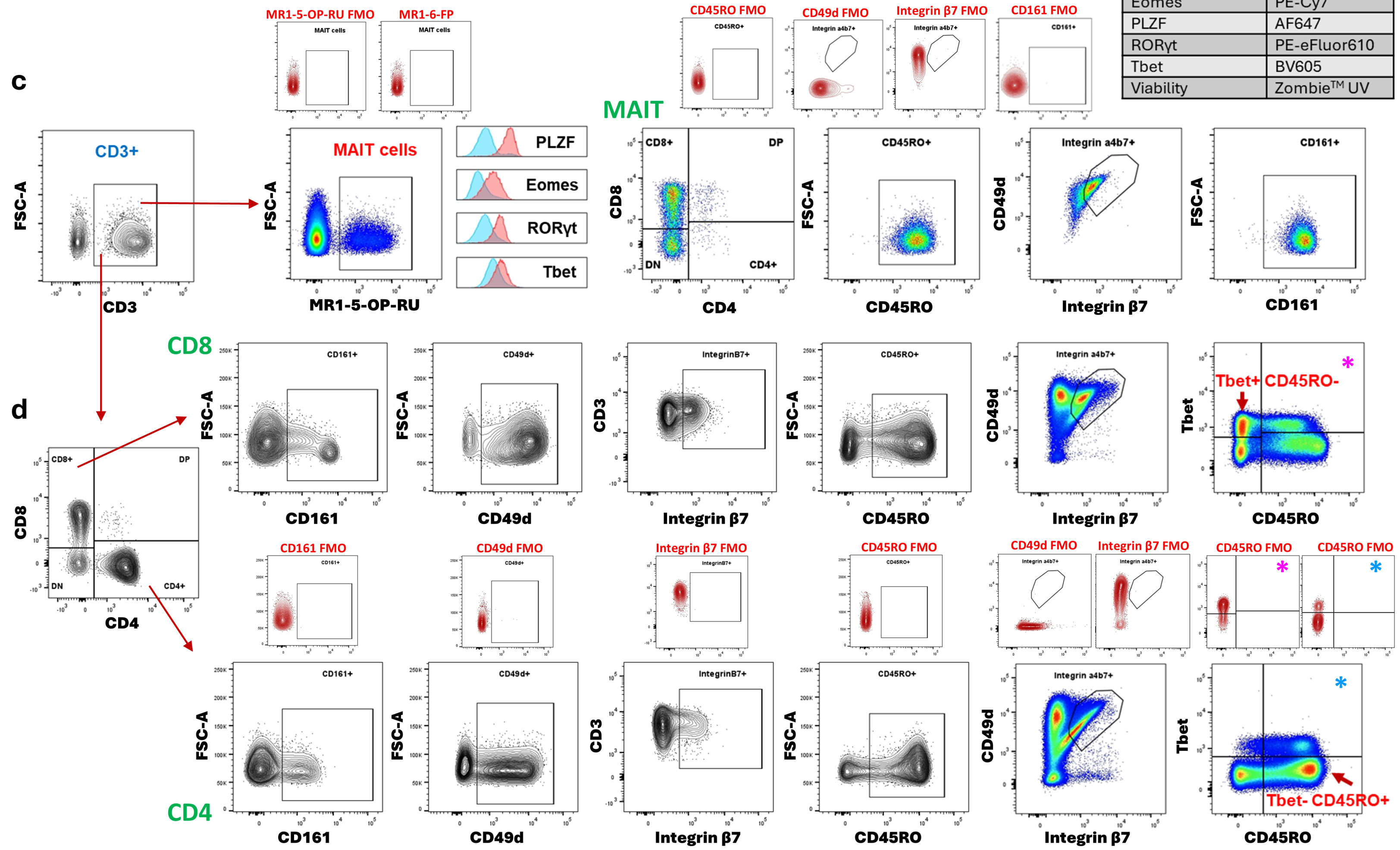

Figure S2. Flow cytometry Panel 2 antibody details and gating strategy *Representative gating for flow cytometry Panel 2, 'MAIT cell characterisation' panel. (a) Preprocessing of flow cytometry events by first eliminating any technical artefacts in the time dimension (if applicable), then gating of live cells, singlets, and lymphocytes based on size. (b) Panel antibody details. (c) MAIT cell gating strategy, gated from CD3+ lymphocytes (T cells). Left: population-gated overlayed histograms of T cells (blue) and MAIT cells (red) demonstrating canonical expression of MAIT-associated transcription factors. Right: marker expression gates for MAIT cells. (d) CD8 (top row) and CD4 (bottom row) T cell gating strategy. Fluorescence minus one (FMO) controls shown as red contour plots above or below corresponding gates or otherwise indicated for the relevant plot with a coloured asterisk (*).*

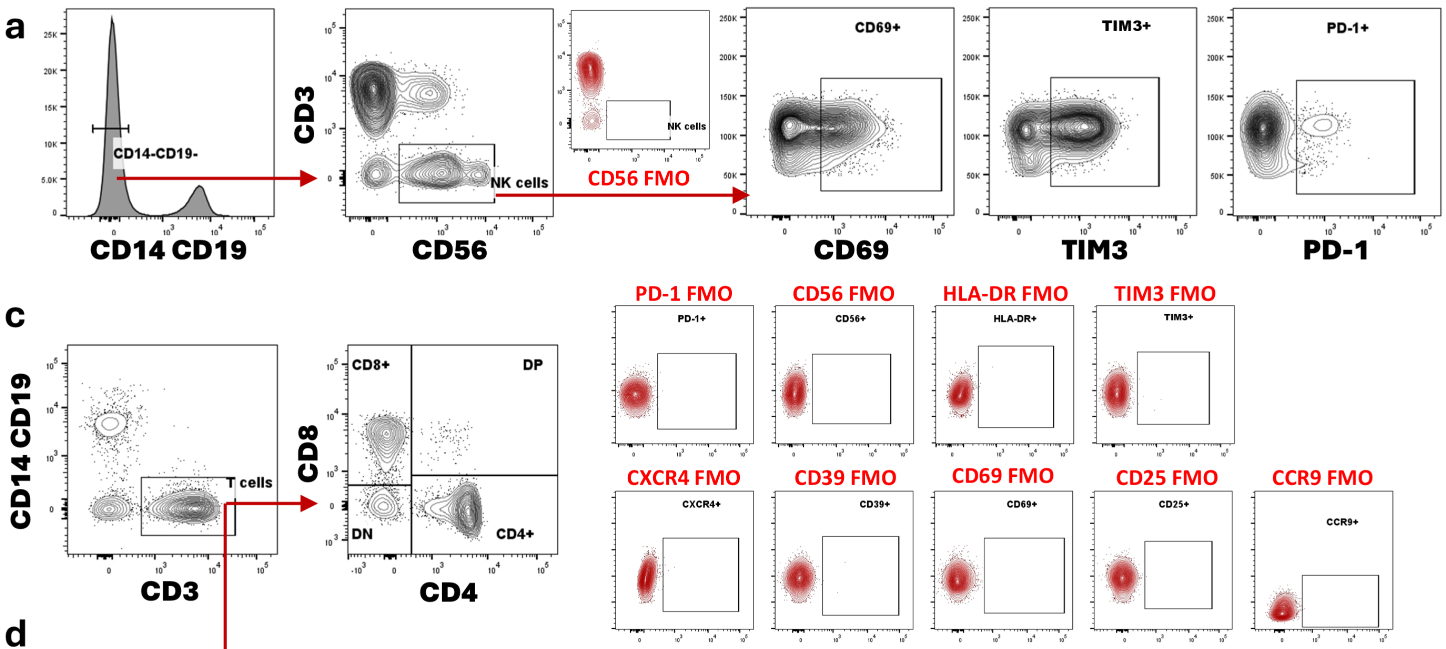

b

Antigen	*Fluorochrome*
MR1-5-OP-RU	PE
CD3	BUV737
CD4	AF700
CD8	APC-H7
CD14	PE-CF594
CD19	PE-CF594
CD25	BV785
CD39	BV605
HLA-DR	KIRAVIA blue
CD69	PE-Cy7
PD-1	AF647
TIM3	BV421
CXCR4	BV510
CCR9	PerCP-Cy5.5
CD56	BUV395
Viability	Zombie™ UV

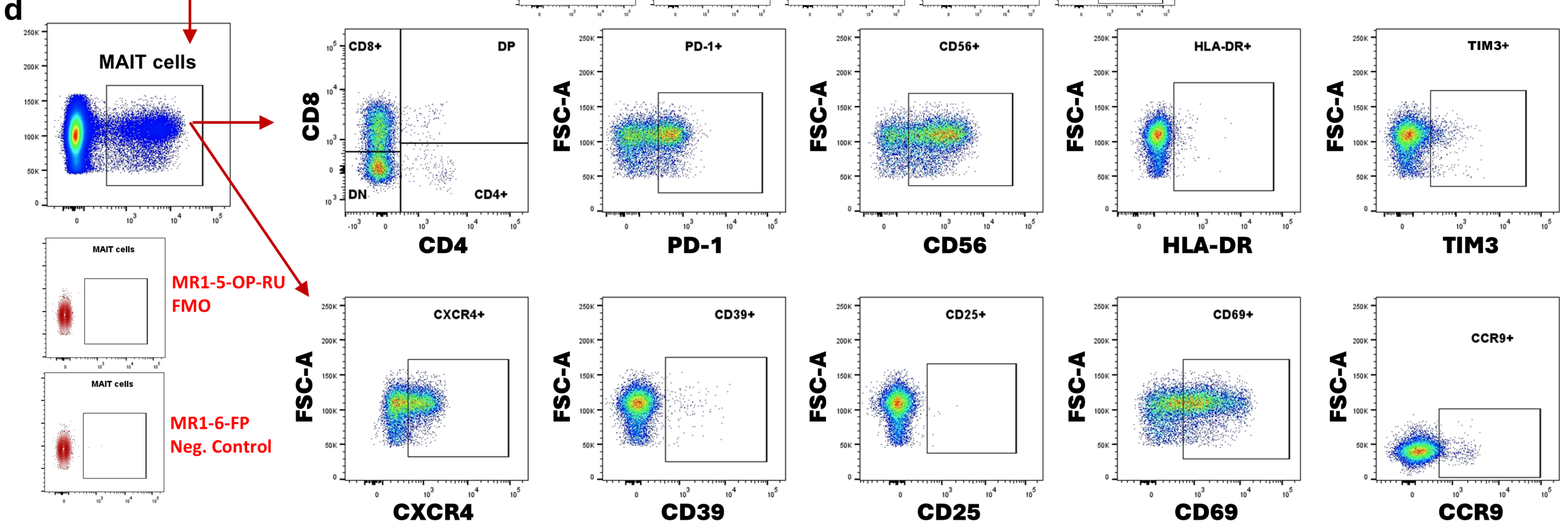

Figure S3. Flow cytometry Panel 3 antibody details and gating strategy. *Representative gating for flow cytometry Panel 3, 'T cell activation markers and NK cells'. (a) Gating of NK cells from pre-processed lymphocytes (see strategy shown in Figure 18A), defined as CD14-CD19-CD3-CD56+. (b) Panel antibody details. (c) Gating of T cells from pre-processed lymphocytes, defined as CD14-CD19-CD3+. (d) Representative MAIT cell gating strategy, from events within the T cell gate. Fluorescence minus one (FMO) controls and MR1-tetramer with 6-formyl pterin (6-FP) negative control shown as red contour plots, from events within the T cell gate.*

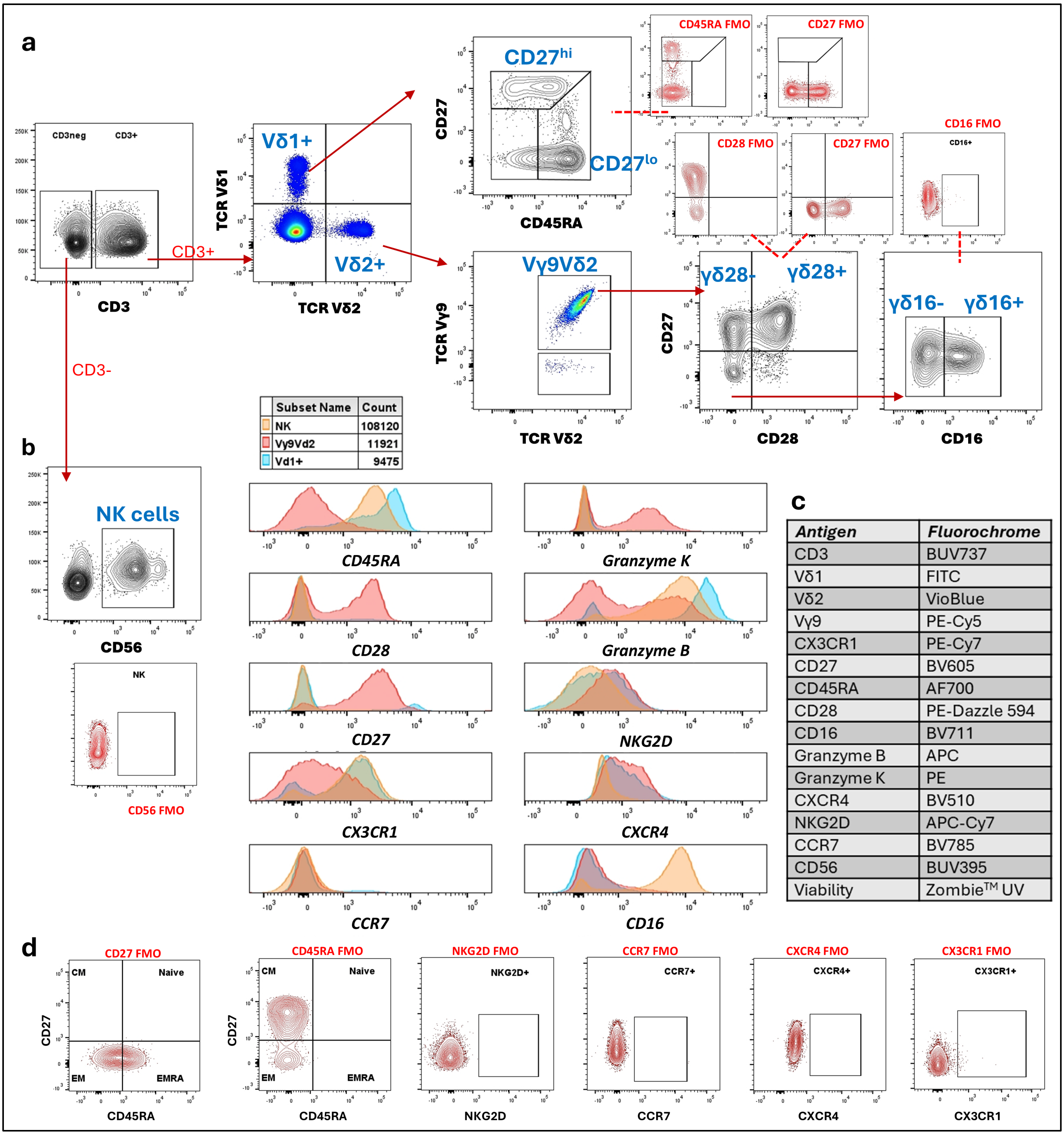

Antigen	Fluorochrome
CD3	BUV737
Vδ1	FITC
Vδ2	VioBlue
Vγ9	PE-Cy5
CX3CR1	PE-Cy7
CD27	BV605
CD45RA	AF700
CD28	PE-Dazzle 594
CD16	BV711
Granzyme B	APC
Granzyme K	PE
CXCR4	BV510
NKG2D	APC-Cy7
CCR7	BV785
CD56	BUV395
Viability	Zombie™ UV

Figure S4. Flow cytometry Panel 4 antibody details and gating strategy. *Representative gating for flow cytometry Panel 4, 'γδ T cell subsets and additional NK markers'. (a) Left: gating of Vδ1 and Vδ2 T cells from pre-processed lymphocytes (see strategy shown in Figure 18A), with Vδ2 T cells defined stringently as Vγ9+Vδ2+ T cells. Right: Gating for Vδ1 T cell CD27^{hi} and CD27^{lo} subpopulations; and Vδ2 T cell γδ28-, γδ28+, γδ16-, γδ16+ subpopulations. (b) Left: NK cell gate, defined as CD3-CD56+ lymphocytes. Right: Representative sample overlayed histograms, qualitatively comparing expression of panel markers among NK cells (orange), Vδ1 T cells (blue), and Vδ2 T cells*

(red). (c) Panel antibody details. (d) Marker expression gate FMO controls, with gate shown for events within Vδ1/Vδ2 T cell gates or T cell gate, depending on number of γδ T cell events within the FMO sample.

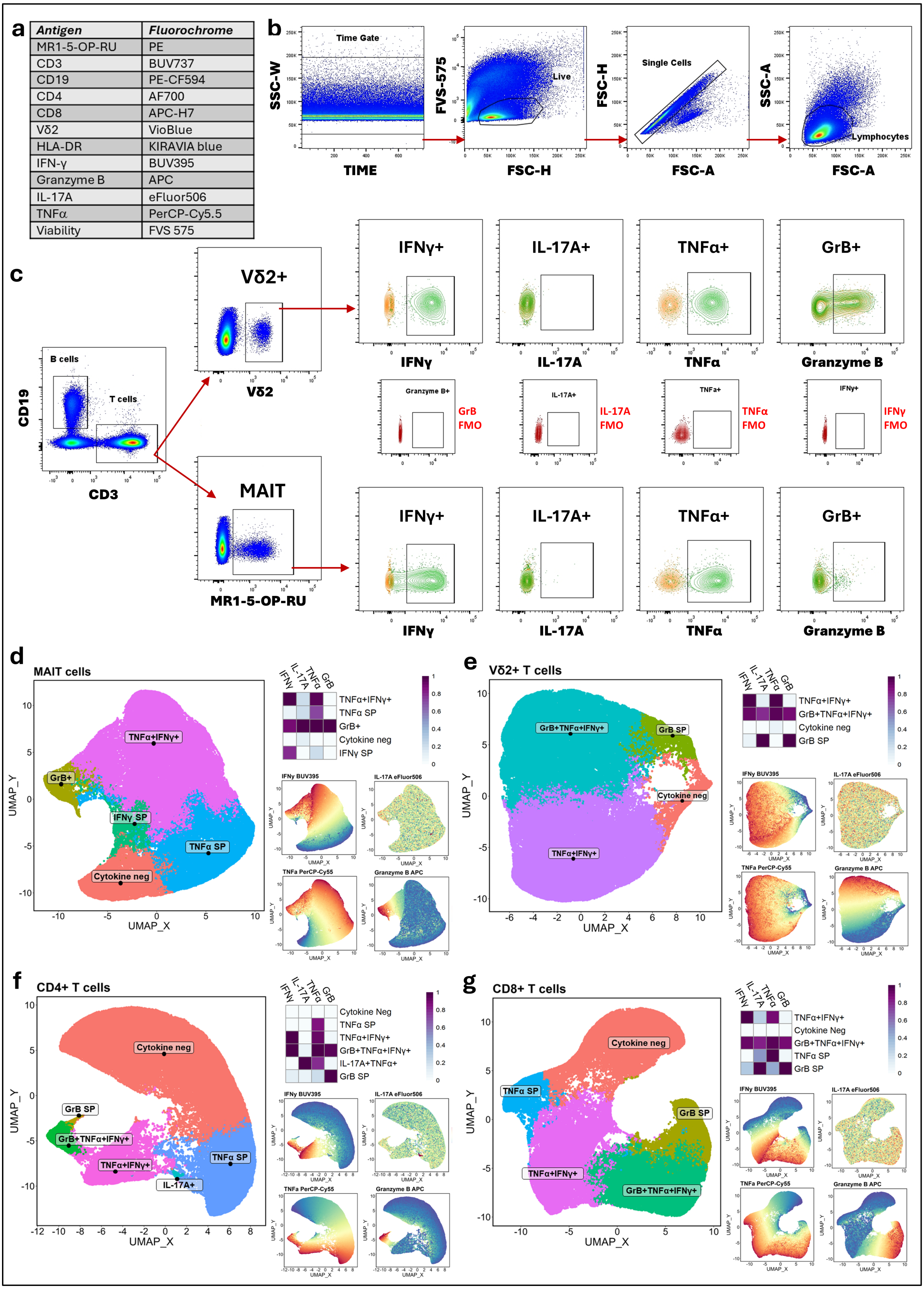

Figure S5. Flow cytometry Panel 5 antibody details and gating strategy. *Representative gating for flow cytometry Panel 5, 'T cell stimulation'. (a) Antibody details. (b) Preprocessing of flow cytometry events by first eliminating any technical artefacts in the time dimension (if applicable), then gating live cells, singlets, and lymphocytes based on size. (c) Gating strategy for MAIT and Vδ2 T cells, with overlayed contour plot data shown for a representative participant's stimulated (green) and unstimulated (orange) samples. Fluorescence minus one (FMO) controls for adjacent marker positivity gates shown as red contour plots. (d-g) Clustering of stimulated MAIT cells, Vδ2 T cells, CD4 T cells, and CD8 T cells, respectively, based on expression of IFNγ, IL-17A, TNFα, and Granzyme B. For each cell type, FlowSOM clustering was performed on the entire dataset, then cluster identities overlayed visually on UMAP performed on a subset of 100,000 cells (50,000 PD; 50,000 HC). Heatmaps showing normalised expression of each marker by individual clusters is shown (top, right), as well as UMAPs coloured according to individual marker expression (bottom, right).*

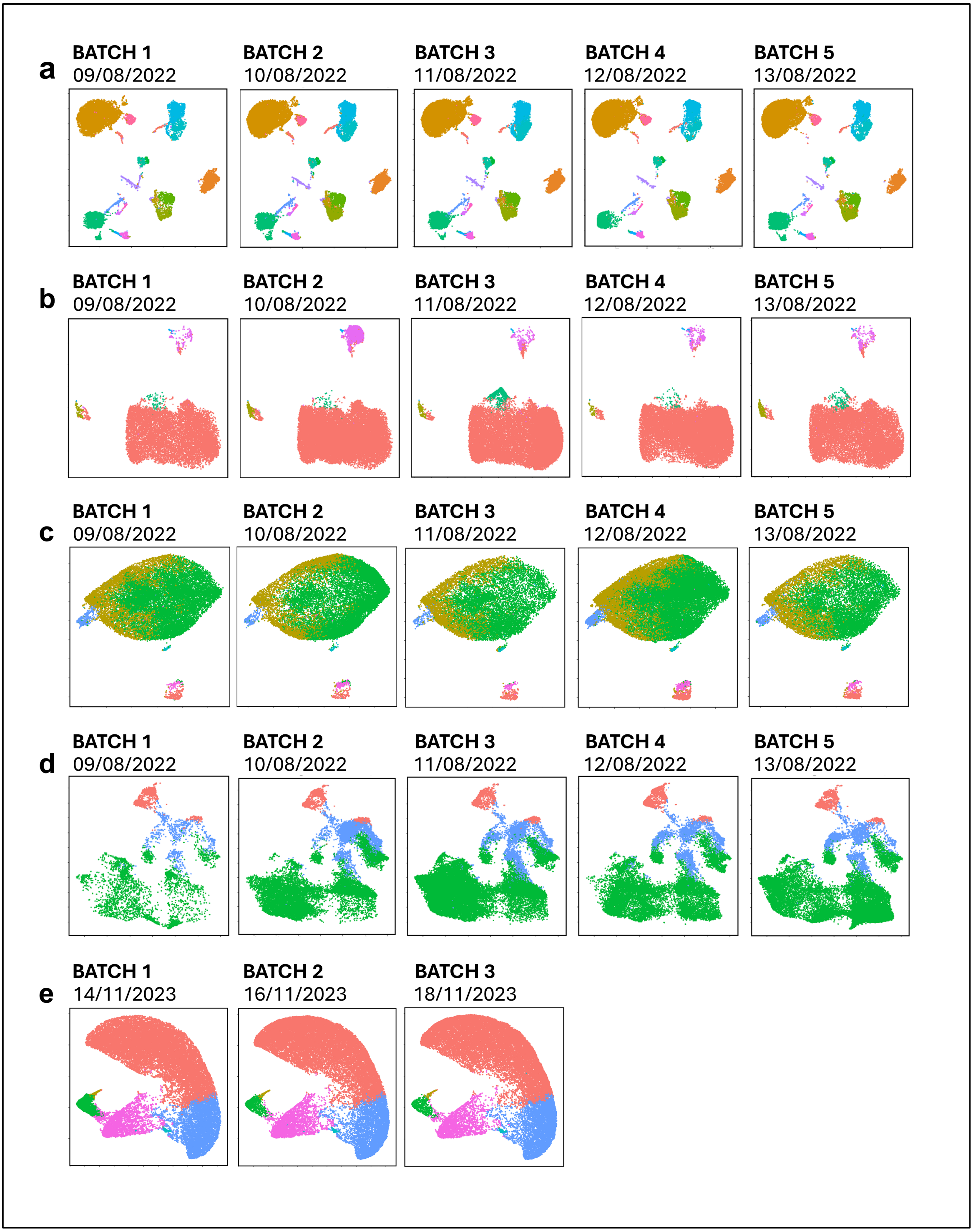

Figure S6. Visualisation of experimental batch integration for flow cytometry panels 1-5. *UMAPs showing division of a 100,000 cell random subset of clustered parameters divided according to experimental batch, with gates chosen randomly for visualisation purposes, including: (a) Panel 1, gated upon live cells; (b) Panel 2, gated upon MAIT cells; (c) Panel 3, gated upon MAIT cells; (d) Panel 4, gated upon Vδ1 T cells; (e) Panel 5, gated upon CD4 T cells.*

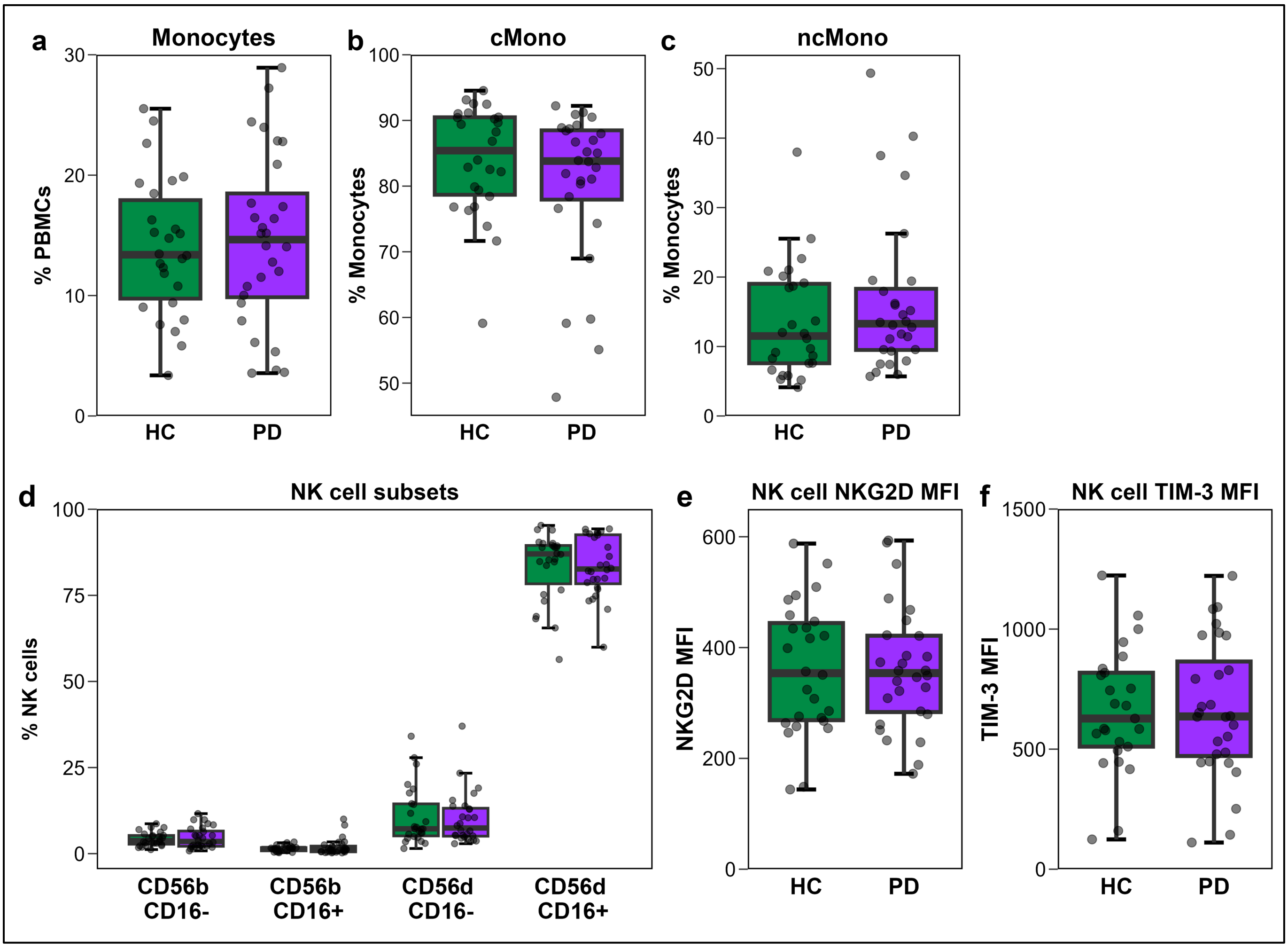

Figure S7. Additional innate immune cell parameters. *(a-d) Groupwise boxplots showing the frequency of: (a) monocytes among live PBMCs; (b) classical monocytes among monocytes; (c) non-classical monocytes among monocytes; and (d) NK cell subsets among NK cells. (e-f) Boxplots showing NK cell expression measured by MFI for markers (e) NKG2D and (f) TIM-3. Boxplots are coloured according to study group (n=25-26 HC, green; n=28 PD, purple).*

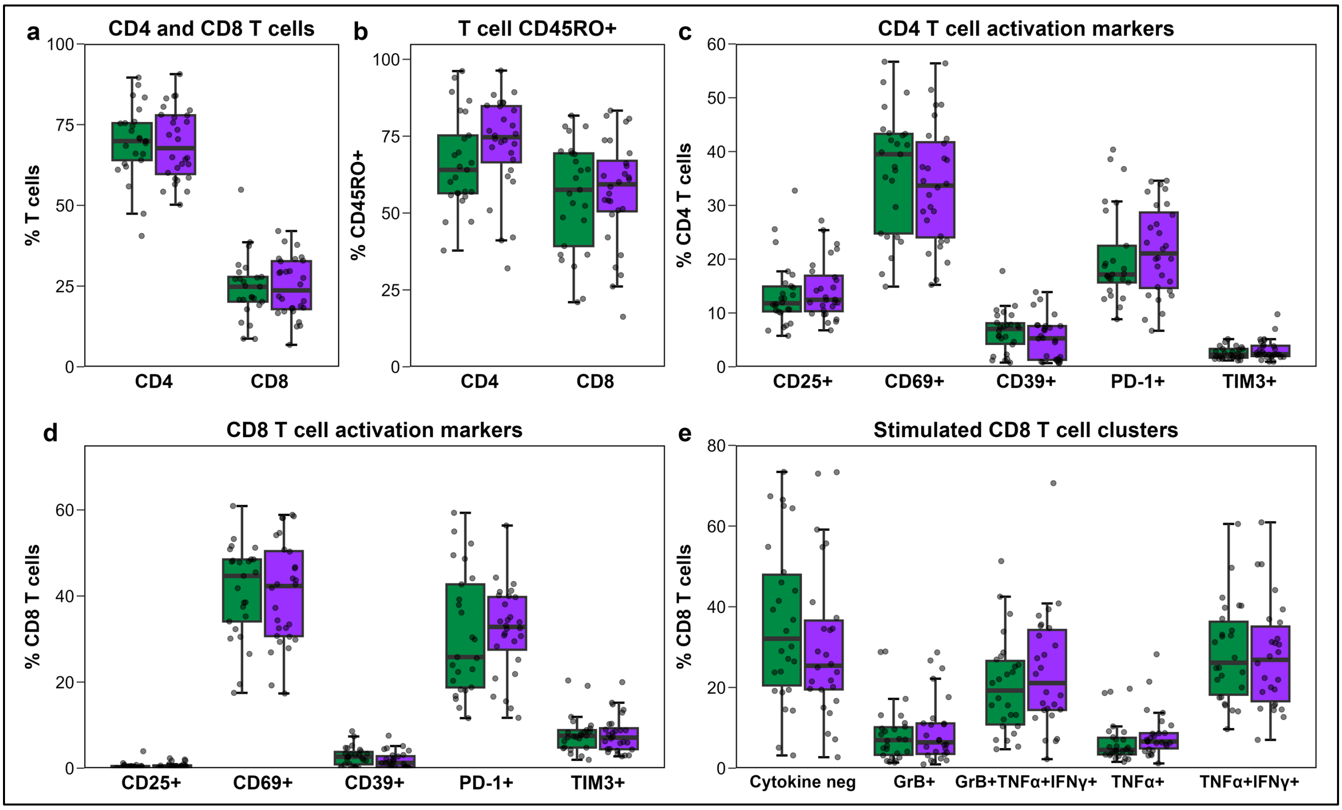

Figure S8. Additional T cell parameters. *(a-e) Boxplots showing the frequency of: (a) CD4 and CD8 T cells; (b) CD45RO+ CD4 and CD8 T cells; (c) CD4 and (d) CD8 T cells expressing activation-associated markers CD25, CD69, CD39, PD-1, and TIM-3; (e) Stimulated CD8 T cell clusters based on expression of granzyme B, TNFα, IFNγ, and IL-17A. Boxplots are coloured according to study group (n=25-26 HC, green; n=26-28 PD, purple).*

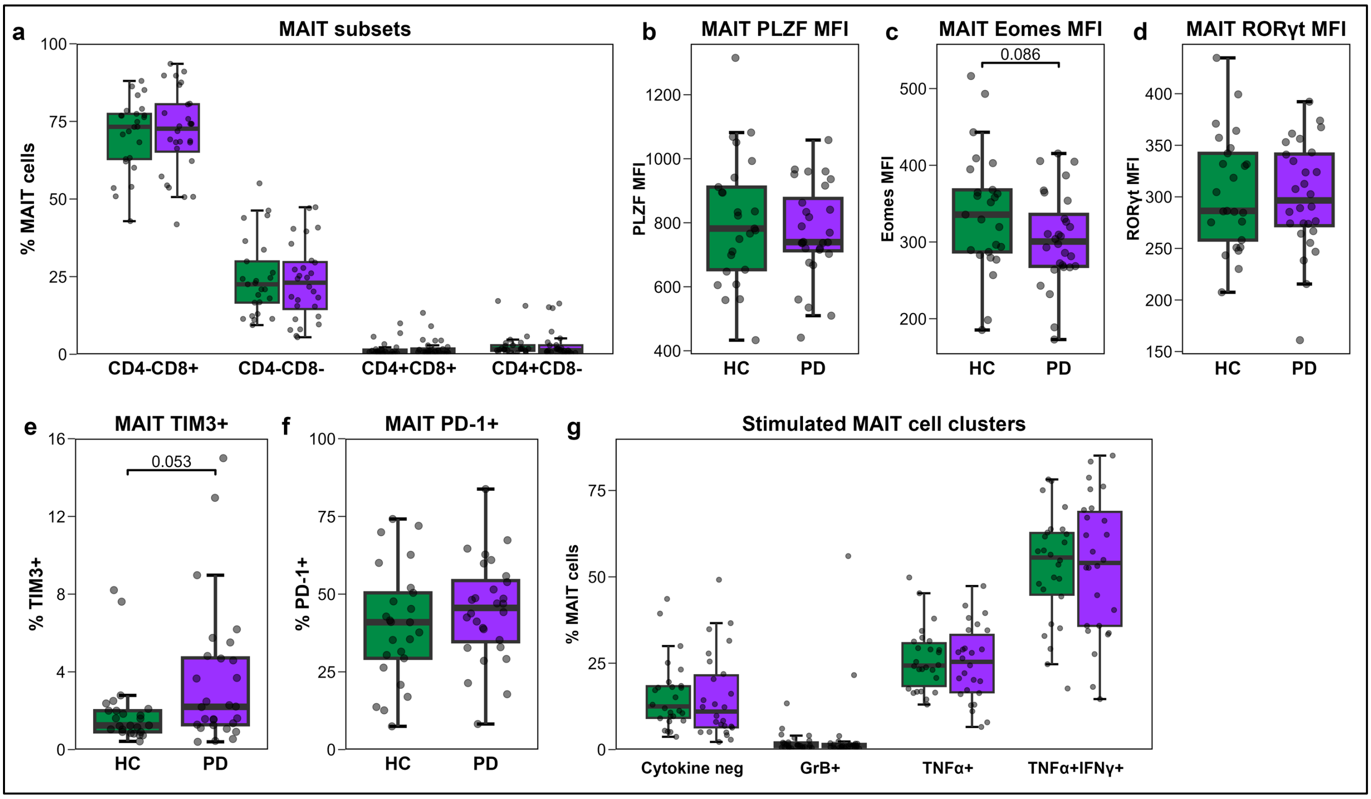

Figure. S9 Additional MAIT cell parameters. *(a-g) Boxplots showing: (a) Frequency of MAIT cell subsets defined by CD4 and CD8 expression; (b) MAIT cell PLZF MFI; (c) MAIT cell Eomes MFI; (d) MAIT cell RORγt MFI; (e) frequency of MAIT cells expressing TIM3; (f) frequency of MAIT cells expressing PD-1; and (g) frequency of stimulated MAIT cell clusters 'cytokine negative', 'granzyme B+', 'TNFα+', and 'TNFα +IFNγ+'. Boxplots are coloured according to study group (n=25-26 HC, green; n=26-28 PD, purple). Statistical comparisons shown were performed by t-test or Wilcoxon rank-sum test, as appropriate.*

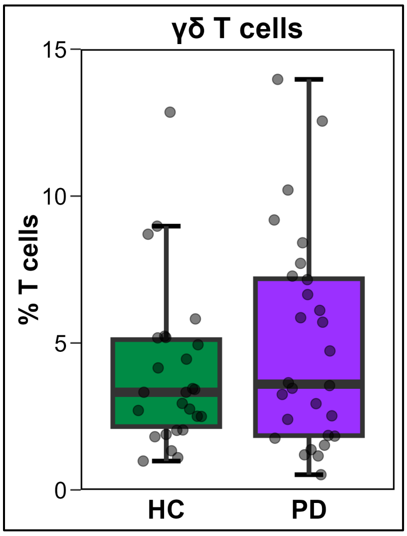

Figure S10. Frequency of γδ T cells among T cells in PwPD (n=28, purple) and HCs (n=26, green).

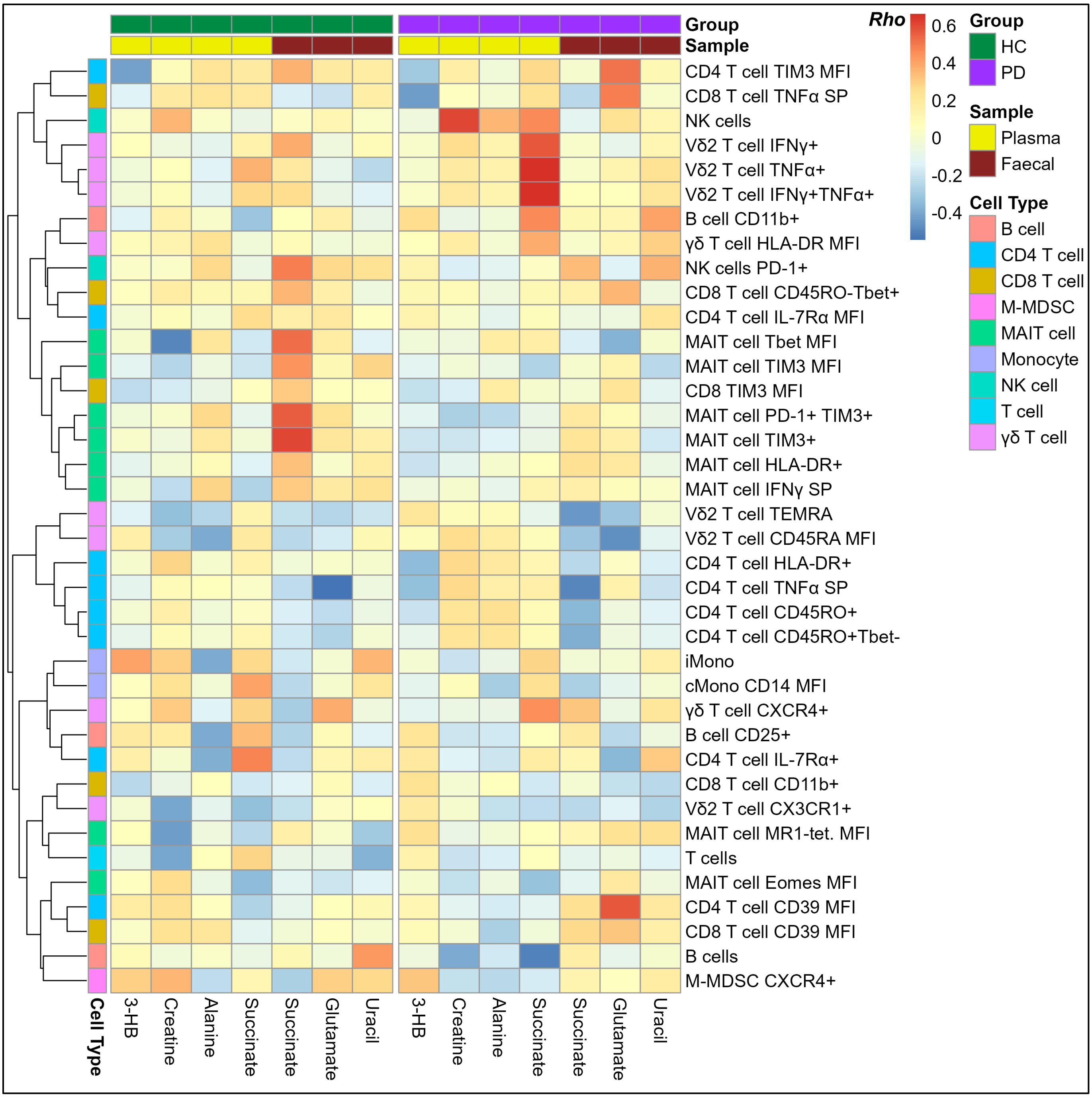

Figure S11. Immune-metabolite correlation coefficient heatmap grouped by disease status. *Heatmap of Spearman's rank correlation coefficients derived from the pairwise correlations between immune (rows) and metabolite measures (columns) identified as associated with PD. Boxes are coloured according to the magnitude of Spearman's rho, ranging from red to blue for relative strong positive (max|R|= 0.61) and negative correlations (max|R| = 0.59), respectively. Columns are coloured according to sample type (plasma: yellow; faeces: brown) and study group (PD: purple; HC: green). Rows are coloured by cell type (see key) and ordered by hierarchical clustering, with dendrogram shown.*

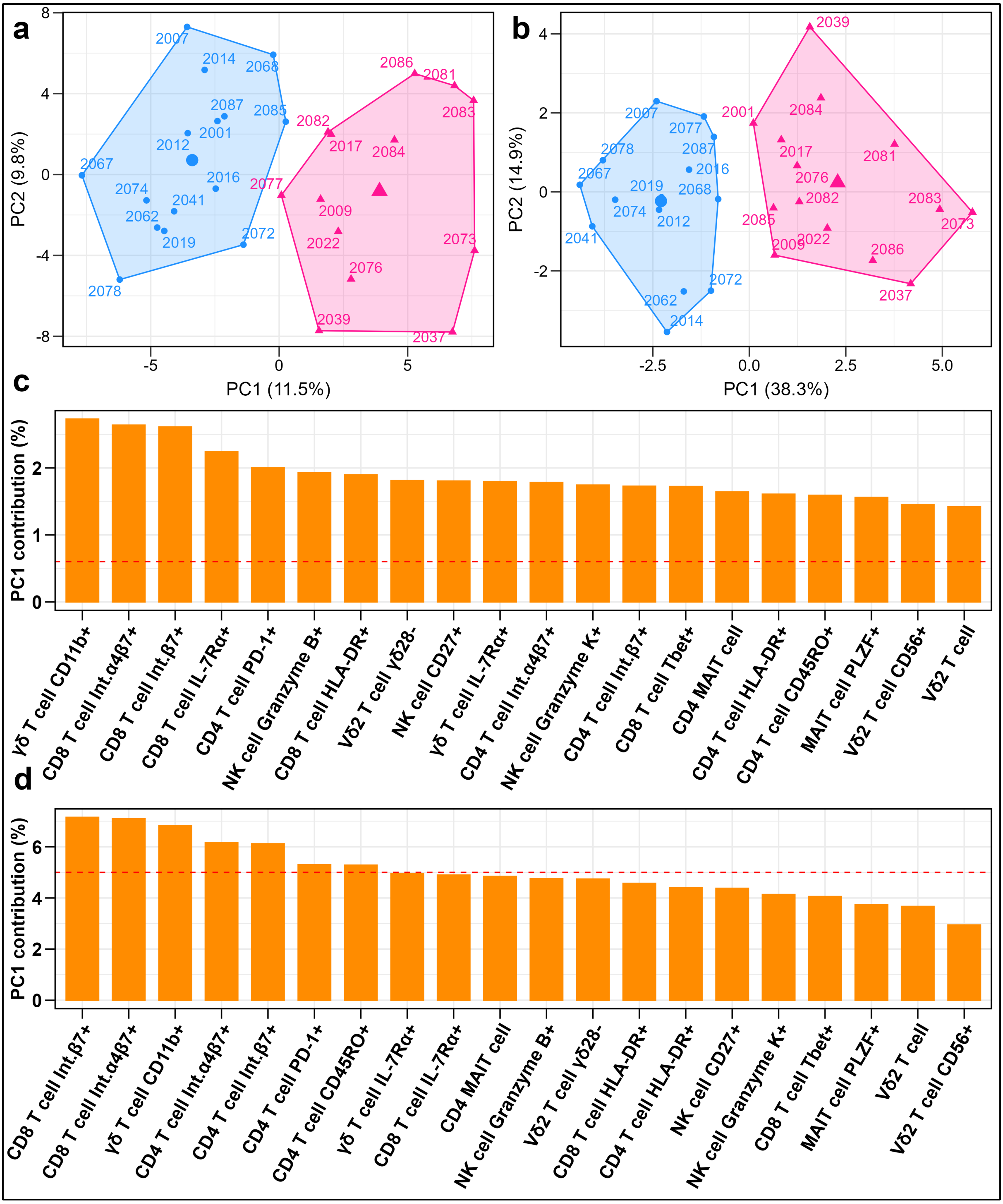

Figure S12. PCA plots and variable contributions with relation to K-means clustering of blood parameters. *(a) PD participants (n=28) were clustered using a dataset of 158 blood immune and metabolite parameters, with K-means (k=2) cluster identities of individuals overlayed on a PCA plot (principal components 1 and 2) generated using this dataset. (b) PCA plot of PD individuals according to the top 20 highest-contributing variables to principal component 1 from the analysis in (A), with re-clustering performed (k=2) and cluster identities overlayed. (c) Bar chart showing percentage contribution of top 20 contributing variables to the first principal component of the PCA analysis performed in (a). (d) Bar chart showing the percentage contribution of variables to the first principal component of the PCA analysis shown in (b). On the bar charts, the red dotted line is indicative of the expected percentage contribution value if the contribution of all variables were uniform.*

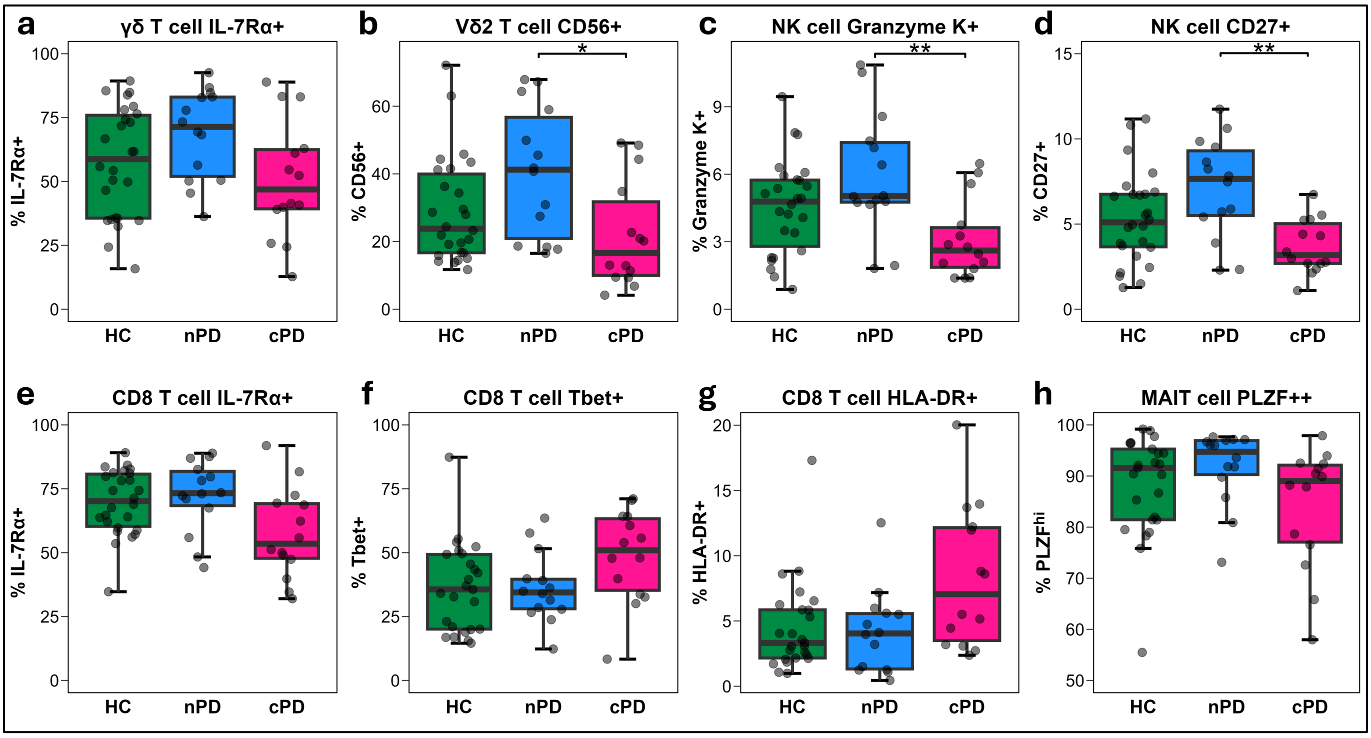

Figure S13. Additional cluster-defining immune variables compared between PD endophenotypes and HCs. *Boxplots showing frequency (among parent cell population) of remaining eight PD cluster-defining immune variables in PwPD grouped according to endophenotype (nPD: blue, n=14; cPD pink, n=14) and HCs (green, n=25-26), including: (a) IL-7Rα+ γδ T cells; (b) CD56+ Vδ2 T cells; (c) granzyme K+ NK cells; (d) CD27+ NK cells; (e) IL-7Rα+ CD8 T cells; (f) Tbet+ CD8 T cells; (g) HLA-DR+ CD8 T cells; and (h) PLZF^{hi} MAIT cells. Between-group comparisons performed using ANOVA or Kruskal-Wallis test, as appropriate. If significant, post-hoc Tukey's HSD or Dunn's test with Bonferroni correction for multiple comparisons was performed, respectively. Statistical significance is indicated as*p<0.05, **p<0.01.*

Table S1. Faecal NMR sub-cohort characteristics. *Values presented as mean +/- standard deviation [range]. Parametric and non-parametric continuous variables were compared using Welch two-sample t-test or Wilcoxon rank sum tests, respectively. Categorical variables were compared using Fisher's exact test.*

		PD (n=24)	HC (n=24)	*p-value*
Age (years)		64 +/- 9 [40 – 83]	64 +/- 10 [37 – 80]	0.97
Sex (M/F)		14/10	9/15	0.25
Gastrointestinal Scale Rating Symptom (GSRS) total score		6.5 +/- 4.0 [1 – 15]	4.5 +/- 3.7 [0 – 12]	0.060
Body Mass Index (BMI, kg/m²)		25.7 +/-3.5 [20.0 – 33.6]	25.1 +/- 3.7 [19.9 – 34.5]	0.57
Age at disease onset (years)		55 +/- 12 [31 – 79]		
Disease duration (years)		8 +/- 8 [0 – 32]		
Hoehn and Yahr Scale	***Stage 1***	12		
	Stage 2	6		
	Stage 3	4		
	Stage 4	2		
UPDRS Part III Score		25.5 +/- 14.7 [8 – 62]		
Levodopa Equivalent Daily Dose (LEDD, mg/day)		829 +/- 733 [0 – 2632.5]		

Table S2. Analysis of additional questionnaire demographic variables. *Differences refer to statistical comparison of PD (n=31) and HC (n=26) participant responses to individual questions from GSRS survey (orange) or study questionnaire (blue). Direction of change (up or down) is with reference to PD subjects compared to HCs. Yes/no question responses and Likert scale variables were analysed using Fisher's exact test and Wilcoxon rank-sum test, respectively. P<0.05*, p<0.01**, p<0.001****

Variable	Change (↑/↓)	p-value	Question additional details
Yes/No variables			
Allergies or intolerances	--	0.421	
Constipation	↑	<0.0001***	
Depression or anxiety	--	0.267	
Reflux	--	0.591	
Current smoking status	--	0.195	
Likert scale variables			
Abdominal distension	--	0.792	Gastrointestinal Symptom Rating Scale (GSRS) (Kulich et al., 2008; Revicki et al., 1998; Svedlund et al., 1988) Each question graded on a 4-point Likert scale (0 – no occurrence of symptom, to 3 – frequent symptom occurrence), and tallied to give a total severity score indicative of overall level of GI dysfunction (Kenna et al., 2021).
Abdominal pains	--	0.091	
Acid regurgitation	--	1.000	
Borborygmus	--	0.831	
Bothered by hard stools	--	0.226	
Bothered by loose stools	--	0.062	
Decreased stool passage	↑	0.006**	
Eructation	--	0.651	
Feeling of incomplete evacuation	↑	<0.001***	
Heartburn	--	0.717	
Increased flatus	--	0.208	
Increased stool passage	--	0.833	
Nausea	--	0.476	
Sucking sensations	--	0.291	
Urgent need to defecate	--	0.560	
Bristol stool scale	--	0.072	Bristol stool scale 1-7 (Blake et al., 2016)
Defecation frequency	↓	0.011*	Less than once/day; once/day; twice/day; 3 times/day; 4 times/day
Alcohol daily consumption	--	0.800	Non-drinker; Yes, once per month; Yes, twice per month; Yes, weekly; Yes, daily
Coffee daily consumption	--	0.373	No/once per week; Yes 1 cup/day; Yes 2 cups/day; Yes, 3cups/day; Yes, 4+ cups/day
Exercise frequency	--	0.489	No/<once per fortnight; Yes, once/week; Yes, twice/week; Yes, every second day; Yes, daily.
Sleep hour category	--	0.406	"How many hours of sleep would you generally get a night? Options: 0-3h, 3-5h, 5-7h, 7-9h, 9+h"
Sleep quality scale	--	0.792	Rated on scale of 1 (poor) to 7 (excellent)
Stress scale	--	0.738	Rated on scale of 1 (no stress) to 7 (extremely stressed)

Table S3. Plasma NMR metabolite concentration data summary. *Metabolite concentrations in are in µM [standard deviation], with fold-change (FC) calculated using group medians. Variables were assessed for normality and between-group comparison performed using a t-test or Wilcoxon rank-sum test, as appropriate, with significant (p<0.05) p-values indicated in bold.*

Metabolite (µM)	HC (n=27)		PD (n=31)		FC	p-value
	Mean [SD]	Median	Mean [SD]	Median		
**3-Hydroxybutyrate**	14 [19]	5.3	5.9 [5.8]	3.9	0.74	**0.039**
**Isobutyrate**	0.97 [0.33]	0.92	0.96 [0.46]	0.9	0.98	0.610
**1-Methylhistidine**	5.5 [0.71]	5.3	5.5 [1.2]	5.3	1.00	0.950
**Acetate**	7.6 [2.7]	6.5	6.9 [1.5]	6.7	1.03	0.734
**Formate**	3.2 [0.72]	3.2	3.4 [0.61]	3.3	1.03	0.338
**Pyruvate**	7.5 [2.2]	7.6	8.4 [3]	8.1	1.07	0.209
**α-Glucose**	400 [80]	390	420 [89]	420	1.08	0.278
**Valine**	29 [6]	27	31 [7.3]	30	1.11	0.176
**Lactate**	250 [77]	250	290 [78]	290	1.16	0.091
**Leucine**	12 [3.2]	12	14 [4.1]	14	1.17	0.164
**Creatine**	6.8 [1.5]	6.9	8.9 [4.8]	8.2	1.19	**0.008**
**Alanine**	30 [6.2]	30	37 [8.9]	36	1.20	**0.004**
**Citrate**	11 [2.8]	10	12 [3.6]	12	1.20	0.297
**Ethanol**	11 [7.7]	8.4	13 [8.2]	11	1.31	0.129
**Isoleucine**	10 [3.1]	9.1	12 [3.8]	12	1.32	0.066
**Tyrosine**	7.2 [2.3]	6.7	10 [3.5]	9.9	1.48	**0.000**
**Succinate**	1.1 [0.75]	0.8	1.8 [1.3]	1.2	1.50	**0.007**

Table S4. Faecal NMR metabolite concentration data summary. *Metabolite concentrations in mM, presented as mean [standard deviation] and median. Fold-change calculated using group medians. Statistical comparison performed by Wilcoxon rank-sum test or t-test, as appropriate, with significant (p<0.05) p values in bold.*

Metabolite (mM)	HC (n=24)		PD (n=24)		FC	p-value
	Mean [SD]	Median	Mean [SD]	Median		
**Succinate**	0.11 [0.076]	0.10	0.077 [0.095]	0.055	0.55	**0.013**
**Trimethylamine**	0.025 [0.048]	0.013	0.010 [0.0079]	0.0089	0.68	0.230
**Uracil**	0.095 [0.029]	0.091	0.072 [0.035]	0.063	0.69	**0.005**
**Glutamate**	1.2 [0.48]	1.1	0.86 [0.37]	0.86	0.78	**0.013**
**α-Glucose**	0.10 [0.073]	0.099	0.16 [0.17]	0.080	0.81	0.830
**β-Glucose**	0.15 [0.13]	0.13	0.25 [0.29]	0.11	0.85	0.862
**Acetate**	5.8 [2.1]	5.3	5.5 [1.4]	5.3	1.00	0.812
**Valine**	0.23 [0.079]	0.21	0.23 [0.057]	0.22	1.05	0.690
**Isoleucine**	0.14 [0.068]	0.13	0.14 [0.035]	0.14	1.08	0.564
**Alanine**	0.38 [0.16]	0.32	0.37 [0.11]	0.35	1.09	0.550
**N-Butyrate**	2.0 [0.99]	1.7	2.1 [1.2]	1.9	1.12	0.628
**Aspartate**	0.21 [0.11]	0.17	0.21 [0.11]	0.19	1.12	0.690
**Propionate**	1.6 [0.52]	1.5	1.8 [0.53]	1.7	1.13	0.270
**Fumarate**	0.022 [0.0099]	0.019	0.026 [0.019]	0.022	1.16	0.877
**2-Hydroxybutyrate**	0.36 [0.12]	0.34	0.38 [0.19]	0.40	1.18	0.310
**3-Hydroxyisobutyrate**	0.55 [0.25]	0.52	0.66 [0.26]	0.62	1.19	0.166
**Formate**	0.016 [0.0097]	0.012	0.092 [0.35]	0.015	1.25	0.392
**Isovalerate**	0.26 [0.13]	0.24	0.31 [0.15]	0.31	1.29	0.215
**Glycerol**	0.38 [0.61]	0.15	0.45 [0.75]	0.26	1.73	0.468

References

Blake, M. R., Raker, J. M., & Whelan, K. (2016). Validity and reliability of the Bristol Stool Form Scale in healthy adults and patients with diarrhoea-predominant irritable bowel syndrome. *Aliment Pharmacol Ther*, *44*(7), 693-703. https://doi.org/10.1111/apt.13746

Kenna, J. E., Bakeberg, M. C., Gorecki, A. M., Chin Yen Tay, A., Winter, S., Mastaglia, F. L., & Anderton, R. S. (2021). Characterization of Gastrointestinal Symptom Type and Severity in Parkinson's Disease: A Case-Control Study in an Australian Cohort. *Mov Disord Clin Pract*, *8*(2), 245-253. https://doi.org/10.1002/mdc3.13134

Kulich, K. R., Madisch, A., Pacini, F., Piqué, J. M., Regula, J., Van Rensburg, C. J., Ujszászy, L., Carlsson, J., Halling, K., & Wiklund, I. K. (2008). Reliability and validity of the Gastrointestinal Symptom Rating Scale (GSRS) and Quality of Life in Reflux and Dyspepsia (QOLRAD) questionnaire in dyspepsia: a six-country study. *Health Qual Life Outcomes*, *6*, 12. https://doi.org/10.1186/1477-7525-6-12

Revicki, D. A., Wood, M., Wiklund, I., & Crawley, J. (1998). Reliability and validity of the Gastrointestinal Symptom Rating Scale in patients with gastroesophageal reflux disease. *Qual Life Res*, *7*(1), 75-83. https://doi.org/10.1023/a:1008841022998

Svedlund, J., Sjödin, I., & Dotevall, G. (1988). GSRS—A clinical rating scale for gastrointestinal symptoms in patients with irritable bowel syndrome and peptic ulcer disease. *Dig Dis Sci*, *33*(2), 129-134. https://doi.org/10.1007/BF01535722
